# Cross-Modal Imaging Reveals Nanoparticle Uptake Dynamics in Hematopoietic Bone Marrow during Inflammation

**DOI:** 10.1021/acsnano.3c11201

**Published:** 2024-02-12

**Authors:** Ashish Tiwari, Narmeen Haj, Betsalel Elgrably, Maria Berihu, Viktor Laskov, Sivan Barash, Shachar Zigron, Hagit Sason, Yosi Shamay, Shiri Karni-Ashkenazi, Maya Holdengreber, Galit Saar, Katrien Vandoorne

**Affiliations:** † Faculty of Biomedical Engineering, Technion-Israel Institute of Technology, Haifa 3200003, Israel; ‡ Biomedical Core Facility, Rappaport Faculty of Medicine, Technion-Israel Institute of Technology, Haifa 3200003, Israel; § Third Faculty of Medicine, Charles University, Prague 100 00, Czech Republic

**Keywords:** iron nanoparticle, inflammation, MRI, intravital microscopy, myelopoiesis

## Abstract

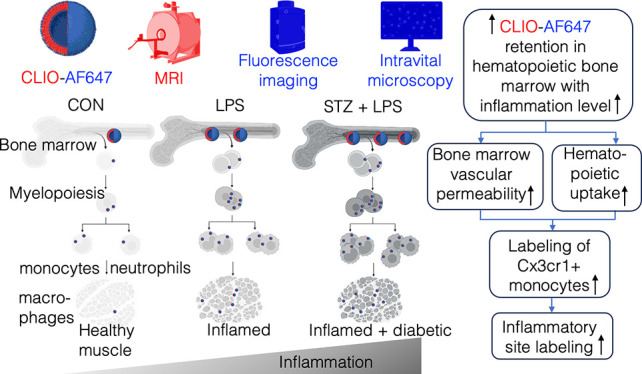

Nanoparticles have been employed to elucidate the innate immune cell biology and trace cells accumulating at inflammation sites. Inflammation prompts innate immune cells, the initial responders, to undergo rapid turnover and replenishment within the hematopoietic bone marrow. Yet, we currently lack a precise understanding of how inflammation affects cellular nanoparticle uptake at the level of progenitors of innate immune cells in the hematopoietic marrow. To bridge this gap, we aimed to develop imaging tools to explore the uptake dynamics of fluorescently labeled cross-linked iron oxide nanoparticles in the bone marrow niche under varying degrees of inflammation. The inflammatory models included mice that received intramuscular lipopolysaccharide injections to induce moderate inflammation and streptozotocin-induced diabetic mice with additional intramuscular lipopolysaccharide injections to intensify inflammation. In vivo magnetic resonance imaging (MRI) and fluorescence imaging revealed an elevated level of nanoparticle uptake at the bone marrow as the levels of inflammation increased. The heightened uptake of nanoparticles within the inflamed marrow was attributed to enhanced permeability and retention with increased nanoparticle intake by hematopoietic progenitor cells. Moreover, intravital microscopy showed increased colocalization of nanoparticles within slowly patrolling monocytes in these inflamed hematopoietic marrow niches. Our discoveries unveil a previously unknown role of the inflamed hematopoietic marrow in enhanced storage and rapid deployment of nanoparticles, which can specifically target innate immune cells at their production site during inflammation. These insights underscore the critical function of the hematopoietic bone marrow in distributing iron nanoparticles to innate immune cells during inflammation. Our findings offer diagnostic and prognostic value, identifying the hematopoietic bone marrow as an imaging biomarker for early detection in inflammation imaging, advancing personalized clinical care.

The immune system and inflammatory processes act as key instigators for a wide spectrum of diseases, ranging from cardiovascular disease, diabetes, and cancer to neurodegenerative diseases.^1−5^ In fact, inflammatory diseases have emerged as the leading cause of death worldwide, accounting for more than 50% of all fatalities.^6^ The inflammatory immune response involves a complex interplay between destructive and reparative processes with innate immune cells such as monocytes, macrophages, and neutrophils playing a pivotal role as first responders in this process. These short-lived innate immune cells are continuously generated by hematopoietic stem and progenitor cells with their production increasing in hematopoietic bone marrow niches during inflammation.^7−9^ Immune responses originating from the hematopoietic system can either be insufficient or overly active, leading to adverse outcomes that negatively impact patients and their chances of recovery.^8,10^ It is worth noting that inflammation and the presence of circulating innate immune cells are closely correlated with mortality rates in sepsis, a condition where the body improperly responds to an infection.^11^ In particular, diabetic patients with enhanced metabolic inflammation face elevated morbidity and mortality linked to sepsis, as compared to nondiabetic patients.^12^

Recent insights regarding the consequences of inflammation^6^ have underscored the clinical need for developing noninvasive techniques capable of accurately and consistently monitoring inflammation from various perspectives. Iron nanoparticles have primarily been employed for magnetic resonance imaging (MRI) of innate immune cells, particularly macrophages, owing to their inherent high endocytosis activity. This has enabled the visualization of leukocytes recruited to local sites of inflammation.^1−4^ Consequently, nanoparticles have found application in inflammation imaging, generating significant clinical interest in various fields, spanning from neurodegenerative diseases^1^ to cardiovascular disease^2,4^ and cancer.^3^ In these specific applications, the utilization of iron oxide nanoparticles holds promise for patient stratification and quantification of treatment efficacy.

Until now, research utilizing methods to diagnose inflammation has concentrated on the local site of inflammation, without considering hematopoietic and immunologic processes at a systems level. In response to systemic inflammatory signals, the local bone marrow environment undergoes changes and transmits incoming information to hematopoietic cells through both surface-bound and secreted signals. This process facilitates either quiescence or proliferation based on the demand for peripheral blood cells.^13^ Given the continuous and high-level recruitment of cells during inflammation, as observed in previous studies,^14,15^ we hypothesize that nanoparticles can be taken up by innate immune cells or even hematopoietic stem and progenitor cells at their production site, which is the hematopoietic bone marrow. This hypothesis is supported by research indicating the clearance of nanoparticles from the blood to hematopoietic organs such as the spleen, lymph nodes, liver, and bone marrow, where accumulation has been observed.^5,16−19^

The dynamics of cellular nanoparticle uptake at the bone marrow in vivo have been explored only to a limited extent due to experimental complexities. Advancements in intravital microscopy at the murine calvaria have allowed us^15,20^ and other researchers^21,22^ to simultaneously track hematopoietic stem and progenitor cells and leukocytes in hematopoietic niches. Additionally, we have demonstrated enhanced permeability and changes in the bone marrow after acute inflammatory stress.^9^ Taking these recent insights into consideration, we extend our hypothesis to propose that nanoparticles not only distribute to innate immune cells within the bone marrow but also accumulate to a greater extent within the hematopoietic marrow in the presence of heightened inflammation. This led us to speculate that nanoparticles can more efficiently label the continuously produced innate immune cells within the hematopoietic marrow during inflammation.

In this preclinical study, our aim was to investigate the dynamics of iron oxide nanoparticles within the hematopoietic bone marrow as inflammatory conditions intensified. These conditions were characterized by an increased level of production and release of innate immune cells. To achieve this, we developed and implemented cross-modal imaging techniques, including MRI, fluorescence imaging, and intravital microscopy, to quantify nanoparticle dynamics at both whole-body and single-cell level. We utilized well-studied cross-linked iron oxide (CLIO) nanoparticles for this purpose.^1,23,24^ Our study involved examining clinically relevant mouse models, including mice subjected to intramuscular lipopolysaccharide injections to induce moderate local inflammation in the muscle. Additionally, we studied diabetic mice induced with streptozotocin, who also received additional intramuscular lipopolysaccharide injections to intensify inflammation.

## Results

### Macrophages Efficiently Phagocytize CLIO Nanoparticles As Imaged by MRI and Fluorescence Imaging

**Figure 1:**
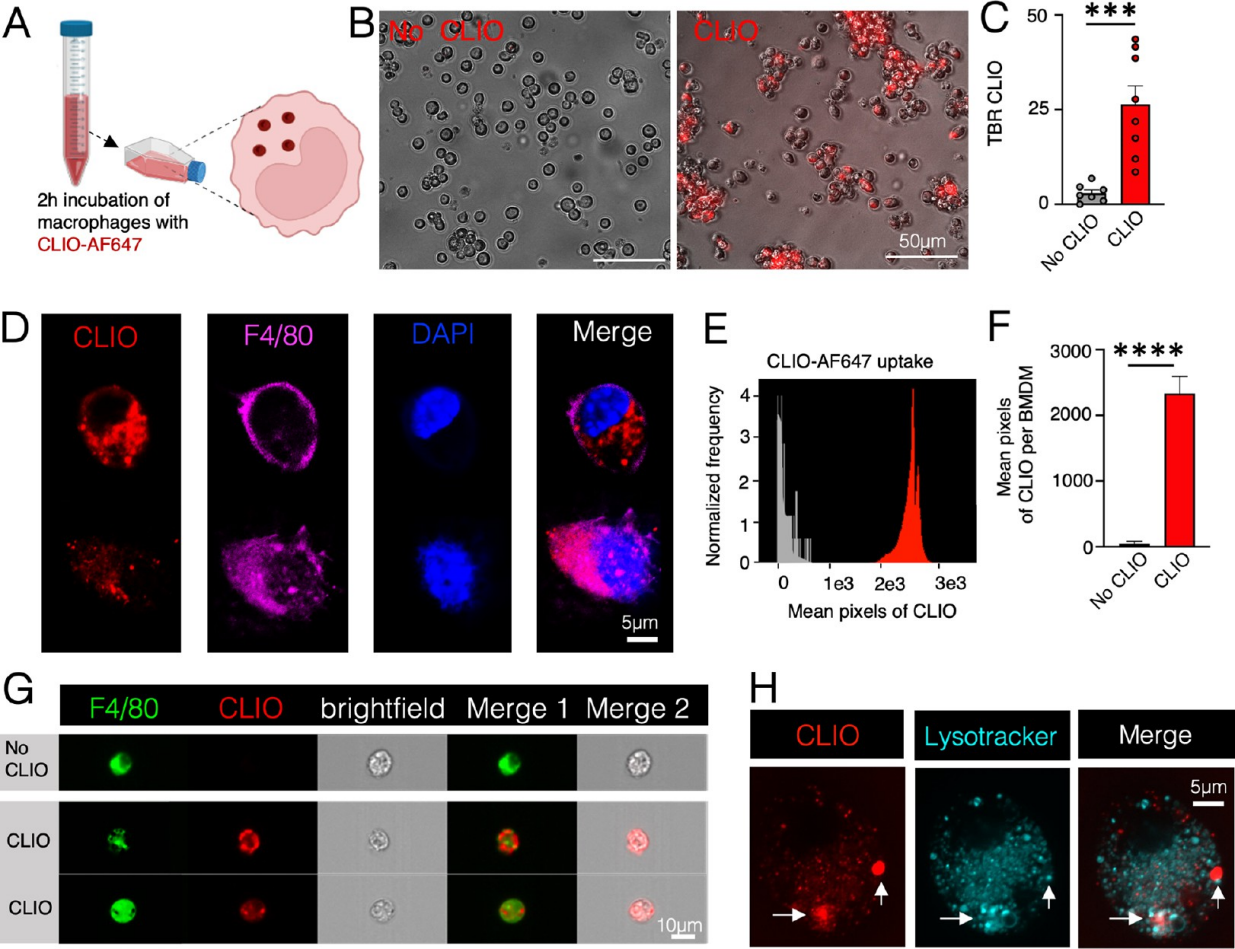
CLIO-AF647 nanoparticles are taken up by cultured macrophages. (A) RAW264.7 macrophages or bone marrow-derived macrophages (BMDMs) were incubated with and without CLIO-AF647 (CLIO) for 2h at 37 °C. (B) Overview microscopic images of RAW264.7 macrophages incubated with or without CLIO. (C) Target-to-background (TBR) fluorescent ratio of CLIO in RAW264.7 macrophages with or without CLIO-AF647 incubation. (D) Confocal microscopy images of RAW264.7 macrophages after incubation with CLIO nanoparticles, showing two representative cells. (E) Histograms and (F) ImageStream flow cytometric quantification of mean pixel of CLIO per BMDM incubated without (gray; *n* = 314) and with (red; *n* = 249) CLIO. (G) ImageStream cytometric visuals display BMDMs in the absence of CLIO (upper panel) and with CLIO nanoparticle internalization (lower panels depict two representative images), with the nanoparticles localized within the cytoplasm of F4/80-positive BMDMs. (H) Confocal micrographs reveal RAW264.7 macrophages post Lysotracker and CLIO nanoparticle treatment. Arrows highlight the colocalization of Lysotracker with CLIO, indicating where nanoparticles fuse with lysosomes (***<0.001, ****<0.0001).

**Figure 2:**
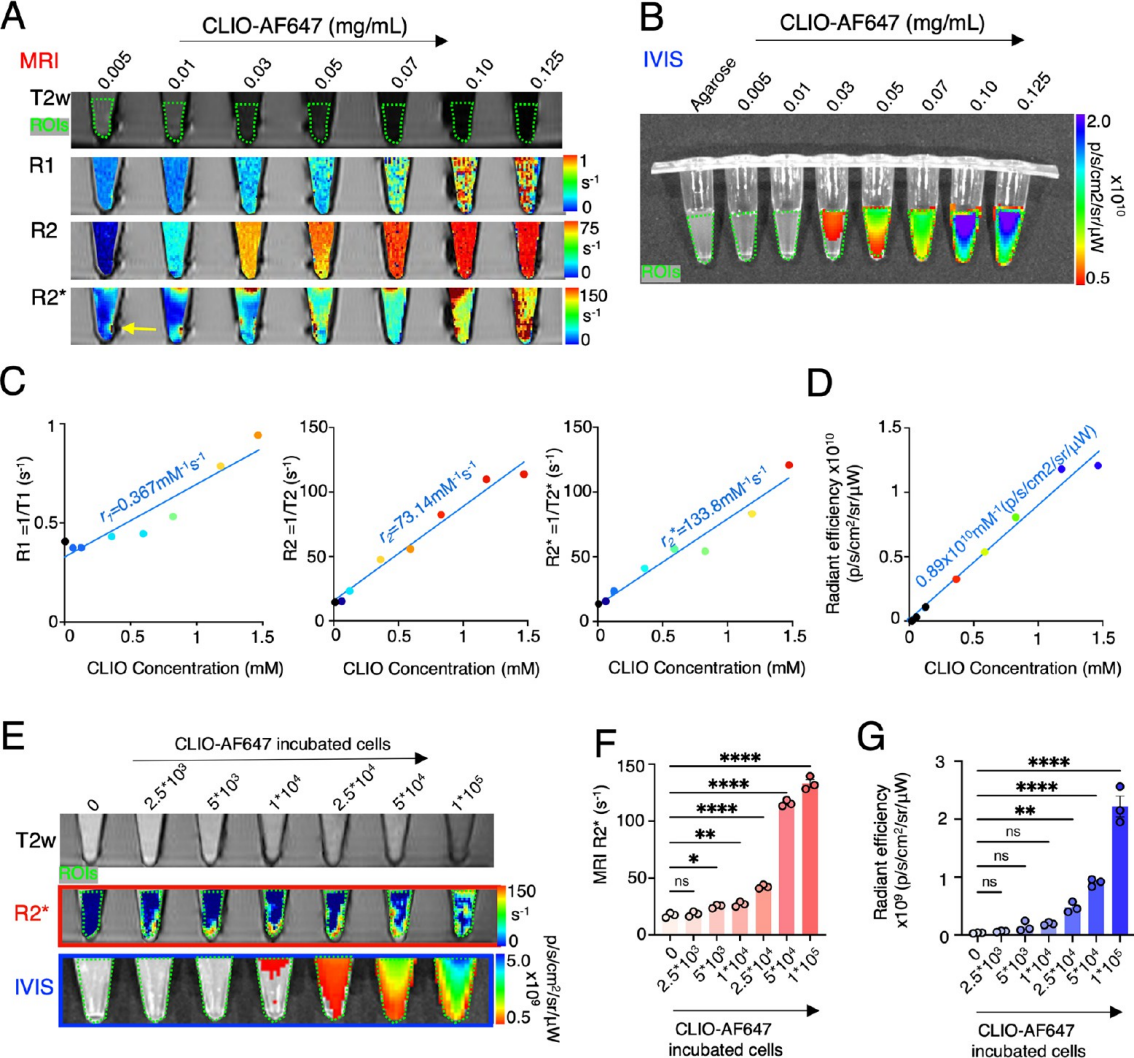
MRI and IVIS fluorescence quantification of signal variations by CLIO-AF647 concentration and by macrophage number incubated with CLIO-AF647. (A) *T*_2_-weighted (*T*_2_w; using Rapid Acquisition with Relaxation Enhancement (RARE) sequence) scout MR image with regions of interest (ROIs) in green dotted line. The rows below are *R*_1_ (by RARE sequence with variable TRs), *R*_2_ (by Multi-Slice Multi-Echo (MSME) sequence with variable TEs), and *R*_2_* (Multi Gradient Echo (MGE) sequence with variable TEs) with parametric maps with increasing concentrations of CLIO-AF647, showing that the lowest number of cells that can be detected with *R*_2_* (yellow arrow). (B) Fluorescence in vivo imaging system (IVIS) images with increasing concentrations of CLIO-AF647 (ROIs in green dotted line). (C, D) Quantification of (C) MRI and (D) IVIS showing an increasing signal with increasing particle concentrations. (E) Phantom *T*_2_w (by RARE sequence), *R*_2_* (by MGE sequence with variable TEs), and IVIS fluorescence images with various numbers of RAW264.7 macrophages after incubation with CLIO-AF647 (ROIs in green dotted line). (F, G) Quantification of signal from (F) *R*_2_* and (G) IVIS fluorescence images in various numbers of macrophages. (MRI was done on a preclinical 9.4T Bruker Biospin; each dot represents one replicate; ns = not significant, *P < 0.05, **P < 0.01, ****P < 0.0001).

The efficacy of Alexa Fluor 647-conjugated CLIO nanoparticles (CLIO-AF647), for labeling innate immune cells, was validated by evaluating the phagocytic uptake of these fluorescently tagged nanoparticles by macrophages. The chemical analysis of CLIO-AF647 nanoparticles showed they possess an average diameter of 76.54 ± 4.00 nm and a zeta potential of −0.84 ± 0.23 mV, aligning with the specifications provided by the supplier, Luna NanoTech (Markham, ON, Canada; Figure S1A, B). Our study utilized RAW264.7 cells, a well-established mouse macrophage line, alongside bone-marrow-derived macrophages (BMDM), which are murine primary bone marrow cells that differentiated and are close to macrophages found in vivo. As these two types of macrophages are innate immune cells and have high phagocytic activity, we chose to investigate them as an initial analysis of nanoparticle uptake for both in vitro and in vivo applications. Either RAW264.7 macrophages or BMDM were cultured in vitro and incubated with CLIO-AF647 nanoparticles for 2h (Figure 1A). We observed that RAW264.7 macrophages engulfed CLIO-AF647 nanoparticles and concurrently expressed macrophage-specific glycoprotein F4/80, indicating successful nanoparticle uptake by macrophages, as revealed by microscopic imaging (Figure 1B–D). Two representative confocal images are shown to illustrate the heterogeneity in nanoparticle uptake (Figure 1D). A cytotoxicity assay was conducted to assess the potential toxicity of the particles to cells in vitro across a range of concentrations over a 2-h period, revealing that cell viability was not affected by CLIO-AF647 particles (Figure S1C). Imagestream cytometric analysis verified the ingestion of CLIO-AF647 nanoparticles by BMDMs, as evidenced by the F4/80 macrophage marker (Figure 1E–G). Two representative Imagestream images are provided to demonstrate the variability in the uptake of CLIO-AF647 nanoparticles (Figure 1G). RAW264.7 macrophages, following incubation with Lysotracker first and CLIO nanoparticles after, displayed intracellular colocalization of the two labels, providing evidence of the nanoparticles’ localization within the lysosomes (Figure 1H). This suggests that macrophages fuse CLIO nanoparticles with lysosomes to sequester and degrade them as part of the body’s immune defense. To optimize imaging methods, we evaluated MRI relaxation rates and fluorescence detectability of CLIO-AF647 nanoparticles that were linearly diluted with agarose and gelified in Eppendorf tubes. Both MRI and fluorescence imaging exhibited high sensitivity in detecting varying concentrations of CLIO-AF647 nanoparticles (Figure 2A–D). Notably, *R*_2_* MRI was capable of detecting the lowest concentration of CLIO-AF647 nanoparticles (Figure 2A). Moreover, the *r*_2_* relaxivity was superior to *r*_1_ or *r*_2_ relaxivity (*r*_2_* = 133.8 mM^–1^ s^–1^ vs *r*_1_ = 0.367 mM^–1^ s^–1^ and *r*_2_ = 73.14 mM^–1^ s^–1^; Figure 2C). Given the enhanced *r*_2_* relaxivity, we proceeded with in vitro and preclinical experiments, focusing exclusively on *R*_2_* mapping by MRI. Subsequent experiments involving increasing numbers of in vitro-loaded macrophages within agarose gel demonstrated a clear, dose-dependent signal enhancement in both *R*_2_* MRI and fluorescence imaging (Figure 2E). Notably, *R*_2_* mapping enabled the detection of 5 × 10^3^ labeled cells in a 100 μL solution, which was significantly different from a 100 μL agarose solution without cells. In comparison, fluorescence imaging exhibited slightly lower sensitivity, detecting 2.5 × 10^4^ labeled cells in the same 100 μL solution compared to solution without cells (Figure 2E–G). The findings validated the feasibility of employing CLIO-AF647 particle imaging for conducting in vivo *R*_2_* MRI and fluorescence imaging studies.

### In Vivo CLIO-AF647 Nanoparticle Uptake by Myeloid Cells in Healthy Mice

**Figure 3:**
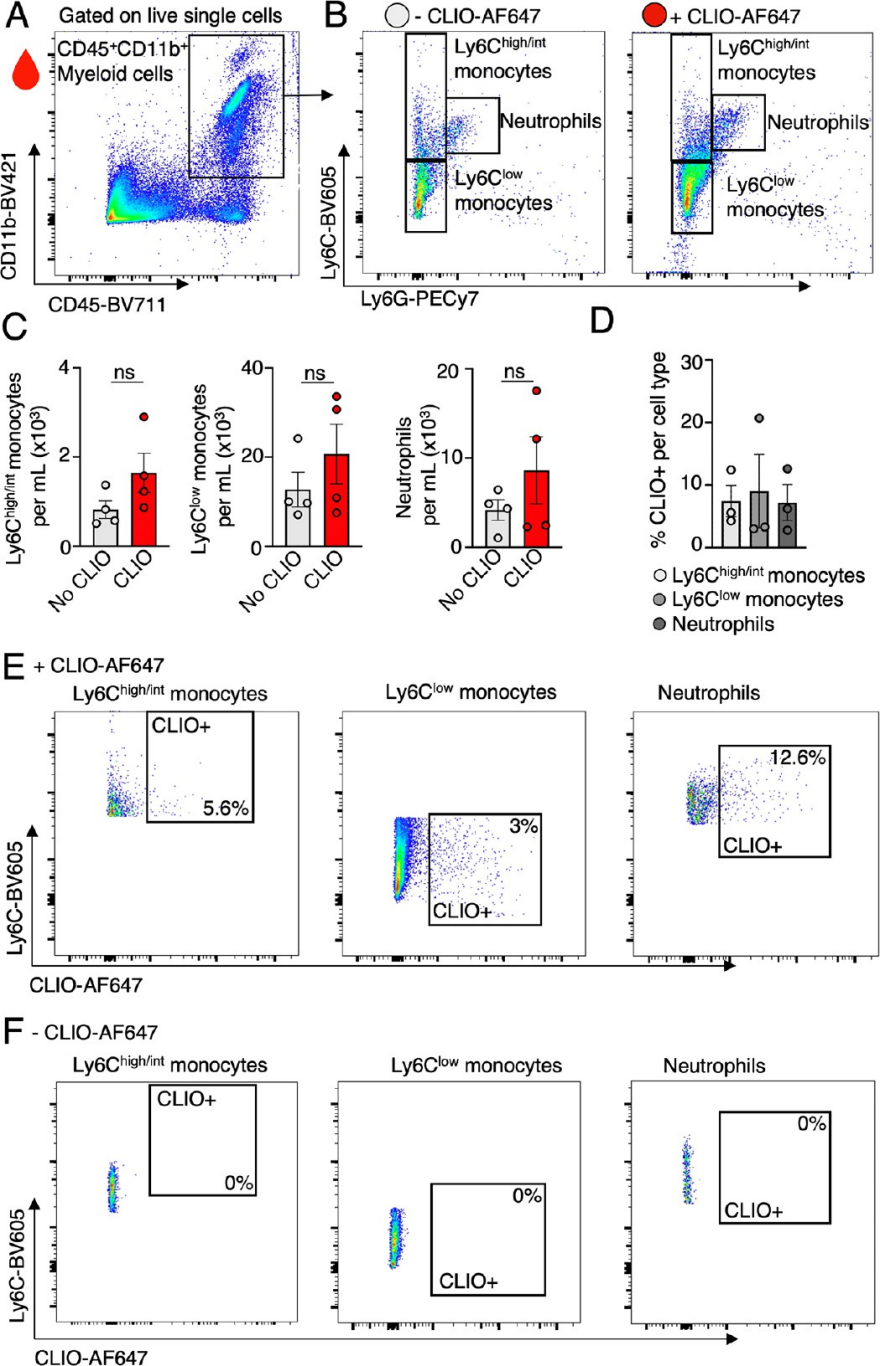
In vivo uptake of CLIO-AF647 nanoparticle by blood myeloid cells in healthy mice. (A) Flow cytometric gating strategy employed to quantify the immune response and nanoparticle uptake 48 h post CLIO-AF647 administration, highlighting CD45^+^CD11b^+^ myeloid cells originating from the bone marrow. (B) Gating strategies to identify distinct myeloid subsets in healthy mice, both with and without CLIO-AF647 administration 48h prior. (C) Numbers of Ly6C^intermediate/high^ (Ly6C^int/high^) monocytes, Ly6C^low^ monocytes, and neutrophils remained unchanged regardless of CLIO-AF647 treatment, indicating no overt immune response elicited by the nanoparticles in healthy mice. (D) Quantitative analysis of CLIO-AF647 uptake within each myeloid subset, with (E, F) representative flow cytometric plots for C^int/high^ monocytes, Ly6C^low^ monocytes, and neutrophils, (E) with and (F) without prior CLIO-AF647 administration.

Myeloid cells, comprising innate immune cells, such as monocytes and neutrophils, travel from the bone marrow through the bloodstream to sites of inflammation. Upon arrival in these tissues, monocytes differentiate into macrophages, executing critical roles such as engulfing pathogens and producing cytokines. To better understand the effect of CLIO-AF647 nanoparticle administration on myeloid cells at a single cell level, we performed flow cytometry analysis on the blood of healthy mice 2 days post-CLIO administration. Blood was stained for CD45, CD11b, Ly6C and Ly6G to differentiate circulating myeloid cells (CD11b^+^CD45^+^) into patrolling monocytes (CD11b^+^CD45^+^Ly6C^low^= Ly6C^low^), inflammatory monocytes (CD11b^+^CD45^+^Ly6C^intermediate/high^ = Ly6C^int/high^), and neutrophils (CD11b^+^CD45^+^Ly6G^+^; Figure 3A, B). Assessing whether CLIO-AF647 administration triggered an immune response, we measured the cell counts of these subtypes 2 days postadministration. We found no significant increase in the number of Ly6C^low^ monocytes, Ly6C^int/high^ monocytes, and neutrophils in the blood with and without CLIO-AF647 administration 2 days prior to this (Figure 3C), indicating no immune activation by the nanoparticles. To pinpoint further the extent to which different myeloid subtypes contribute to nanoparticle uptake, we investigated CLIO nanoparticles uptake in these cells. In the blood, both Ly6C^low^monocytes and Ly6C^int/high^ monocytes internalized the nanoparticles after 2 days, as did neutrophils, with no notable differences in the uptake levels among these cells (Figure 3D–F).

### Nanoparticle Uptake in the Inflamed Marrow Correlates with Inflammation Severity

**Figure 4:**
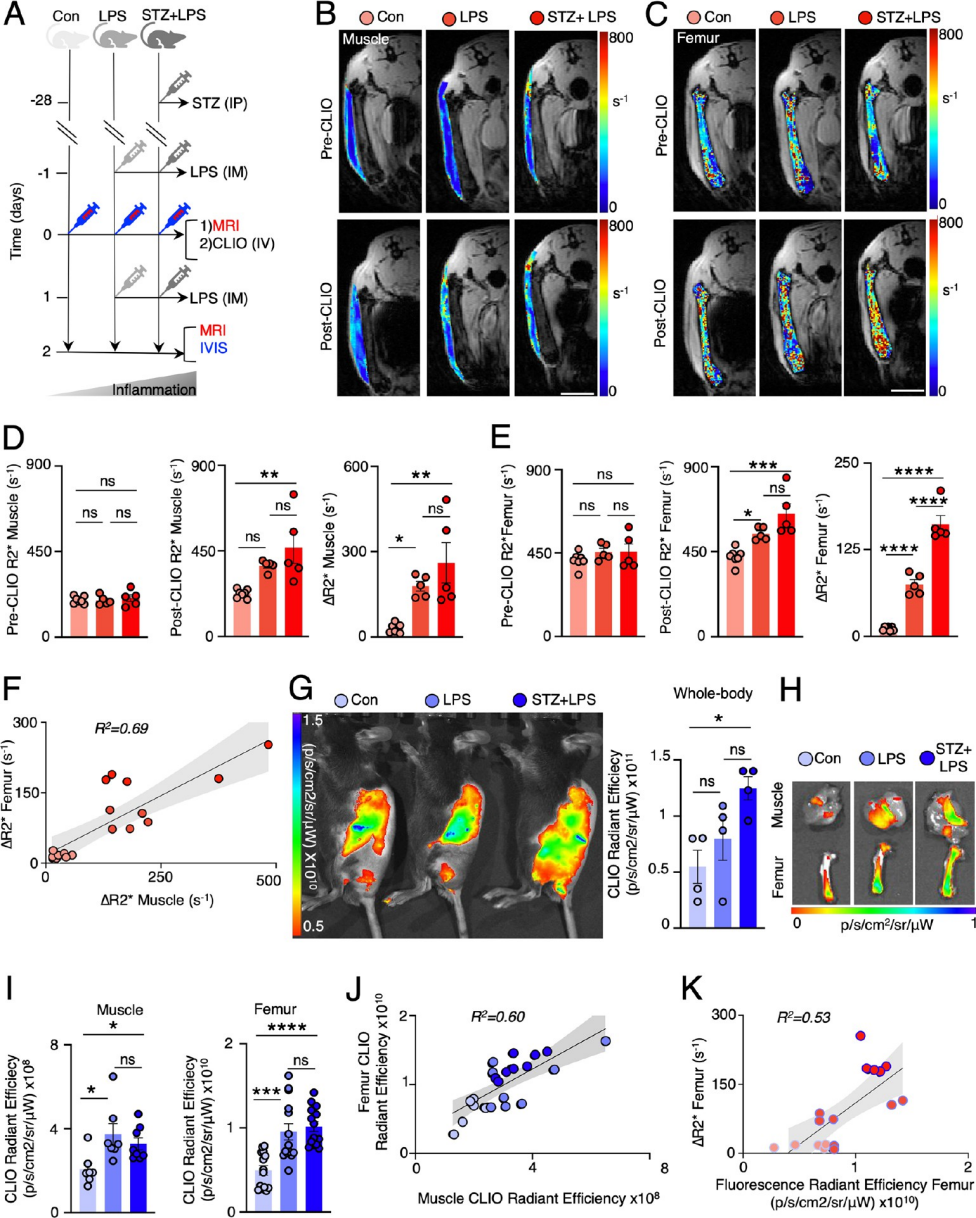
In vivo imaging demonstrates increased uptake of CLIO-AF647 nanoparticles in inflamed muscle and bone marrow. (A) In vivo experimental groups depicted progressive increments in inflammation. All mice, including the controls (Con), were subjected to MRI *R*_2_* imaging before and after MRI/IVIS fluorescence imaging 2 days following the intravenous (i.v.) injection of CLIO-AF647 nanoparticles. Lipopolysaccharide was administered intramuscularly (i.m.) 1 day before and 1 day after the i.v. administration of CLIO-AF647 nanoparticles in normal mice (LPS) and in mice with streptozotocin-induced diabetes (STZ+LPS). (B, C) Representative MRI images with an overlay of *R*_2_* map of (B) muscle and (C) femoral bone marrow (using MGE sequence with variable TEs; scale bar = 1 cm). (D, E) Quantification of pre-CLIO *R*_2_*, post-CLIO *R*_2_*, and Δ*R*_2_* (post-CLIO *R*_2_* minus pre-CLIO *R*_2_*) values in muscle and femoral bone marrow, showing increased signal from CLIO-AF647 nanoparticles with increasing inflammation in muscle and bone marrow. (F) Correlation of Δ*R*_2_* in muscle with Δ*R*_2_* in femoral marrow. (G) In vivo whole-body IVIS fluorescence imaging and quantification of i.v. injected CLIO-AF647 nanoparticles. (H) Ex vivo fluorescence imaging and (I) quantification of CLIO-AF647 nanoparticles in inflamed muscle and bone marrow. (J) Correlation of fluorescence in muscle with fluorescence in femoral marrow. (K) Correlation of Δ*R*_2_* in femoral marrow with fluorescence in femoral marrow. (MRI was done on a preclinical 9.4T Bruker Biospin; Each dot represents one mouse; *P < 0.05, **P < 0.01, ****P < 0.0001).

We next studied whether heightened levels of inflammation would result in varying levels of nanoparticle uptake in both muscle and femoral bone marrow. To conduct this analysis, we utilized high-field 9.4T MRI for *R*_2_* mapping and fluorescence imaging via an in vivo imaging system (IVIS), and we categorized three groups of mice with gradual increasing inflammation. Healthy control mice (Con) were compared to mice injected with lipopolysaccharide intramuscular in the vastus lateralis muscle 1 day before and 1 day after intravenous administration of nanoparticles (LPS). Lastly, diabetic mice, induced by streptozotocin 4 weeks prior, displayed increased blood glucose and higher neutrophil levels than healthy control before LPS injections (Figure S2A). These diabetic mice subsequently received two intramuscular injections of lipopolysaccharide (STZ+LPS), leading to the highest level of inflammation compared with the other experimental groups (Figure 4A). Before nanoparticle administration, there were no detectable differences in *R*_2_* values observed in the muscle and femoral bone marrow among the groups with varying levels of inflammation. To specifically monitor innate immune cells recruited to the inflamed muscle and assess the dynamics of nanoparticle-laden innate immune cells during an increase in inflammation, we intravenously injected CLIO-AF647 nanoparticles.

Two days following the administration of CLIO-AF647, an increased recruitment of CLIO-AF647 nanoparticles taken up by innate immune cells was indeed detected by heightened *R*_2_* values at the inflamed muscles of the LPS and STZ+LPS groups compared to the control muscle. In these muscles, the change in *R*_2_* values before and after CLIO-AF647 injection (Δ*R*_2_*) demonstrated a rise in the LPS and STZ+LPS groups relative to that of control mice, suggesting an infiltration of myeloid cells carrying CLIO-AF647 nanoparticles. In the bone marrow, the analysis of *R*_2_* values before and after nanoparticle injection (Δ*R*_2_*) showed increased Δ*R*_2_* values in the marrow of the LPS and STZ+LPS groups when compared to the control marrow. A notably greater increase in Δ*R*_2_* values was observed in the femoral bone marrow of the LPS and STZ+LPS groups compared to the control group (Figure 4B**–**E; Figure S2B).

Our observations indicated a correlation between Δ*R*_2_* values in the muscle and bone marrow in mice with increasing levels of inflammation. This correlation suggests that CLIO-AF647 uptake in inflamed muscle is associated with CLIO-AF647 uptake in inflamed bone marrow. Fluorescence IVIS imaging demonstrated an increased whole-body signal of CLIO-AF647 in STZ+LPS mice relative to that of the control group. This rise in fluorescence intensity across the entire body evaluation indicates a systemic inflammatory response in the STZ+LPS model. In LPS-treated mice, fluorescence imaging did not exhibit an enhanced uptake of CLIO-AF647 throughout the body (Figure 4G). These whole-body fluorescence imaging data are complementary to the lower limb MRI, particularly due to MRI *R*_2_* mapping’s increased sensitivity which allows for more precise quantification of deeper tissues like bone marrow and muscles located deeper within the body. After whole-body imaging, organs were harvested for ex vivo analysis. Two days after CLIO-AF647 administration, free particles were eliminated from the circulation, as previously documented,^1,5^ and accumulated in the spleen, liver, kidney, and bone marrow (Figure S2C, D). Ex vivo fluorescence analysis confirmed a notable increase in CLIO-AF647 uptake in both the muscle and femoral bone marrow of the LPS and STZ+LPS groups compared with the signal observed in the control muscle and marrow (Figure 4H). Indeed, we observed a correlation between fluorescence imaging values and the gradual increase in inflammation levels, as evidenced by the accumulating levels of nanoparticles in both the muscle and bone marrow as inflammation intensified (Figure 4I, J). We further found a moderately positive correlation (*R*^2^ = 0.53) between the fluorescence signal in femoral bone marrow and MRI signal, suggesting that fluorescence imaging offers a semiquantitative assessment of nanoparticle distribution in alignment with MRI findings (Figure 4K). The moderate correlation observed may be attributed to the inherent limitations of fluorescence imaging in quantifying exact amounts, such as signal overlap, nonlinear response at high concentrations, and background fluorescence interference that can obscure precise measurements.^25^

### CLIO Nanoparticles Colocalize with Cx3cr1^GFP/+^ Monocytes in the Inflamed Bone Marrow

**Figure 5:**
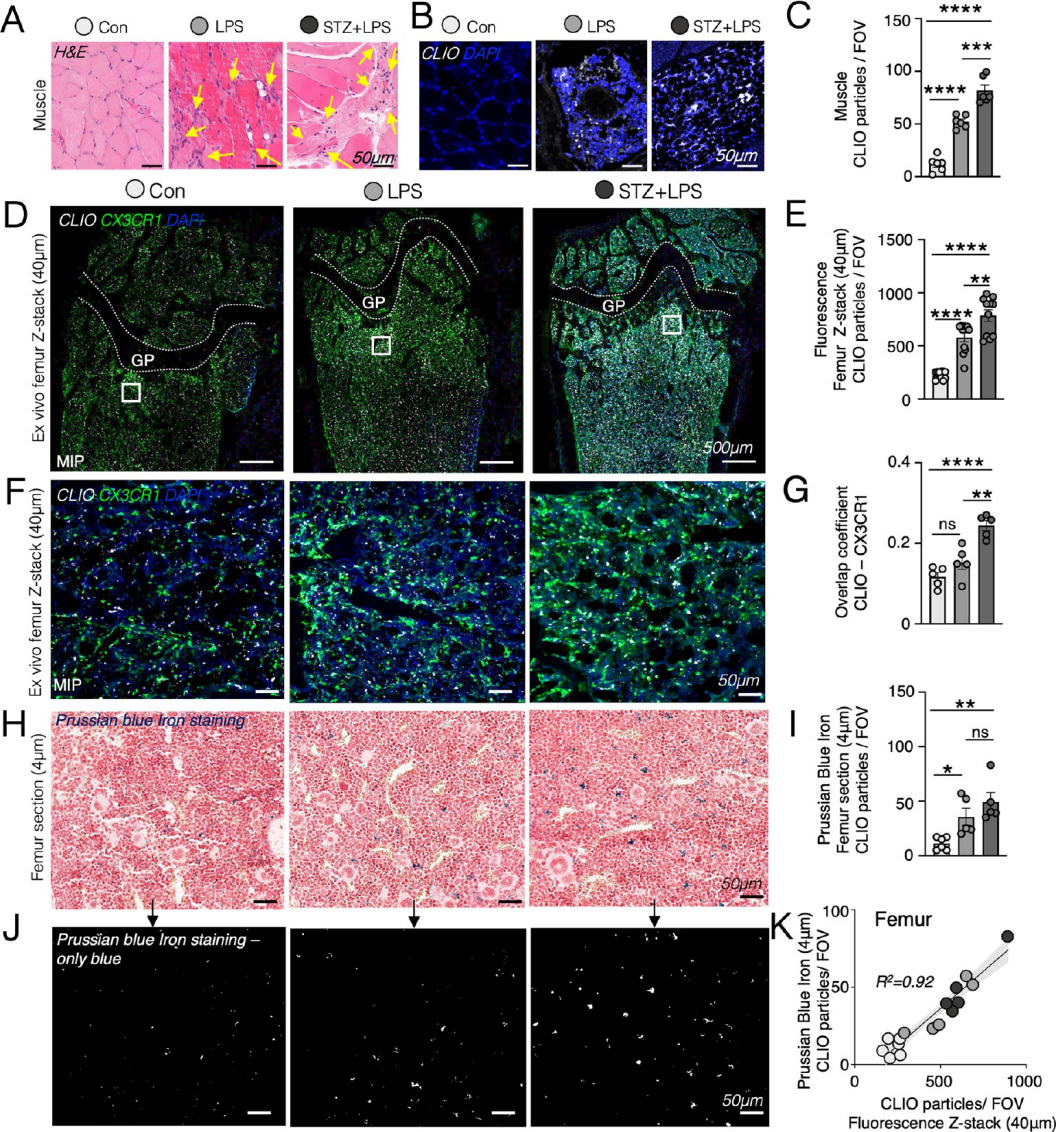
Ex vivo microscopy confirms higher levels of CLIO-AF647 nanoparticles in inflamed muscle and bone marrow. (A) Histological hematoxylin and eosin (H&E) images of muscle tissue, with muscle damage and inflammatory infiltrates indicated by yellow arrows. (B) Fluorescence microscopic images of adjacent muscle tissue showing CLIO-AF647 infiltration (4 μm slice shown). (C) Quantification of CLIO-AF647 particles in muscle per field of view (FOV = 250 × 250 × 4 μm). (D) Confocal maximal intensity projection (MIP) images displaying CLIO-AF647 in Cx3cr1^GFP/+^ femoral bone marrow localizing the FOV below the growth plate (GP) in the metaphysis. (E) Quantification of CLIO-AF647 particles in femoral bone marrow per FOV (250 × 250 × 40 μm). (F) Confocal microscopy images, covering 40 μm depth displaying femoral bone marrow uptake of CLIO-AF647, colocalizing with Cx3cr1^GFP/+^ monocytes and macrophages. (G) Quantification of overlap coefficient between CLIO and Cx3cr1^GFP/+^ monocytes and macrophages in the femoral bone marrow. (H) Perls’ Prussian Blue staining of femoral bone marrow on 4 μm histological sections. (I) Quantification of iron in femoral bone marrow per FOV(250 × 250 × 4 μm). (J) Blue channel of Perls’ Prussian Blue staining to visualize iron deposits from CLIO-AF647. (K) Correlation of CLIO-AF647 fluorescence on 40 μm confocal microscopy images with blue iron deposits of CLIO-AF647 on Perls’ Prussian Blue staining of contralateral femoral bone marrow on 4 μm histological sections (each dot represents one mouse; *P < 0.05, **P < 0.01, ***P < 0.001, ****P < 0.0001).

To complement our in vivo findings and to overcome the limitations of fluorescence imaging, we quantified the number of CLIO-AF647 nanoparticles in both muscle and bone marrow through ex vivo confocal microscopy and histology. H&E staining of the left vastus lateralis muscle revealed muscular damage and the infiltration of inflammatory cells after lipopolysaccharide injection in the LPS and STZ+LPS muscle (Figure 5A). Fluorescent microscopy of the muscle tissue confirmed a significant increase in the number of CLIO-AF647 nanoparticles in the inflamed LPS and STZ+LPS muscle in comparison to the quantity of particles in the control muscle (Figure 5B, C). Furthermore, 2 days after the administration of CLIO-AF647 nanoparticles, bone marrow from the LPS and STZ+LPS groups showed an increased accumulation of nanoparticles, as determined by fluorescent quantification using confocal microscopy with maximum intensity projection (MIP), spanning a 40 μm depth (Figure 5D, E; Figure S3A). Moreover, we observed a greater overlap coefficient between CLIO-AF647 nanoparticles and Cx3cr1^GFP/+^ cells^26^ in the STZ+LPS femur compared to the control and LPS-inflamed femora (Figure 5G). Perls’ Prussian Blue staining of femoral bone marrow confirmed enhanced nanoparticle presence in LPS and STZ+LPS bone marrow on 4 μm sections (Figure 5H–J). Z-stack confocal microscopy findings of the CLIO-AF647 fluorescence signal highly correlated (*R*^2^ = 0.92) with Prussian Blue quantification of iron deposits in the femoral bone marrow sections (Figure 5K).

### Inflamed Bone Marrow Displayed Enhanced Vascular Permeability

**Figure 6:**
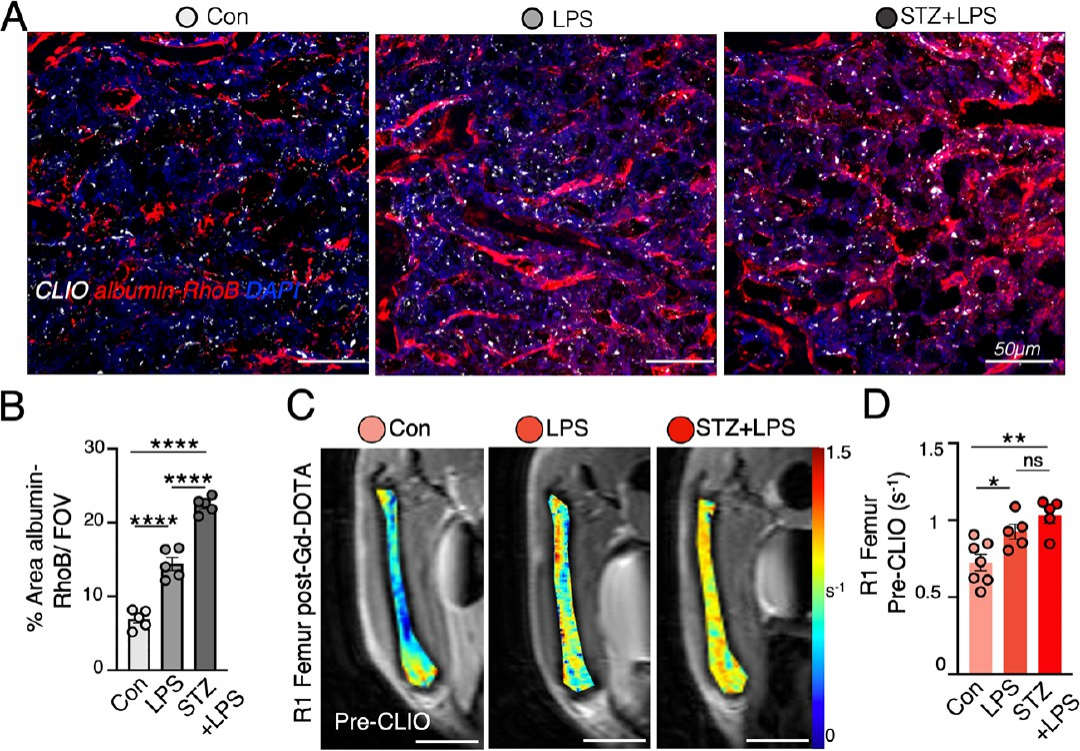
Enhanced permeability at inflamed bone marrow. (A) Confocal microscopy images display femoral bone marrow uptake of CLIO-AF647, colocalizing with albumin-RhoB, a blood pool agent 5 min after injection, which quantifies permeability*surface area of vessels. (B) Percentage (%) area of albumin-RhoB at the femoral bone marrow. (C) Representative *R*_1_ parametric maps of pre-CLIO *R*_1_ after injection of Gadolinium (Gd)-DOTA (using a RARE sequence with variable TRs), showing enhanced permeability and retention of Gd in LPS and STZ+LPS femoral marrow. (D) Quantification of pre-CLIO *R*_1_. (MRI was done on a preclinical 9.4T Bruker Biospin; each dot represents one mouse; *P < 0.05, **P < 0.01, ****P < 0.0001).

In response to LPS-induced inflammation, innate immune cells exit the bone marrow as first responders, partly due to increased blood vessels.^15^ To assess vessel permeability, we administered bovine serum albumin labeled with rhodamine B (albumin-RhoB), a blood pool agent, 5 min before euthanizing the mice. The percent area covering albumin-RhoB per field of view (FOV) was measured, depicting the permeability × surface area product (permeability). An elevated percent area of albumin-RhoB, indicating increased permeability, was observed in both LPS and STZ+LPS bone marrow compared to the control marrow (Figure 6A, B). Furthermore, a gadolinium chelate, Gd-DOTA (Dotarem), was used to characterize permeability at the bone marrow before CLIO injection. In the context of permeable vessels and edema, an enhanced *R*_1_ signal after the administration of Gd-DOTA can suggest that permeable vessels allow the Gd-DOTA to permeate into tissue.^27^ Analysis of post-Gd-DOTA *R*_1_ mapping before CLIO injection confirmed a higher increase in *R*_1_ after contrast enhancement, signifying a greater enhanced permeability and retention (EPR) effect in STZ+LPS inflamed marrow compared to control marrow (Figure 6C, D).

### Elevated CLIO Uptake in Bone Marrow Erythro-Myeloid Progenitors during Inflammation

**Figure 7:**
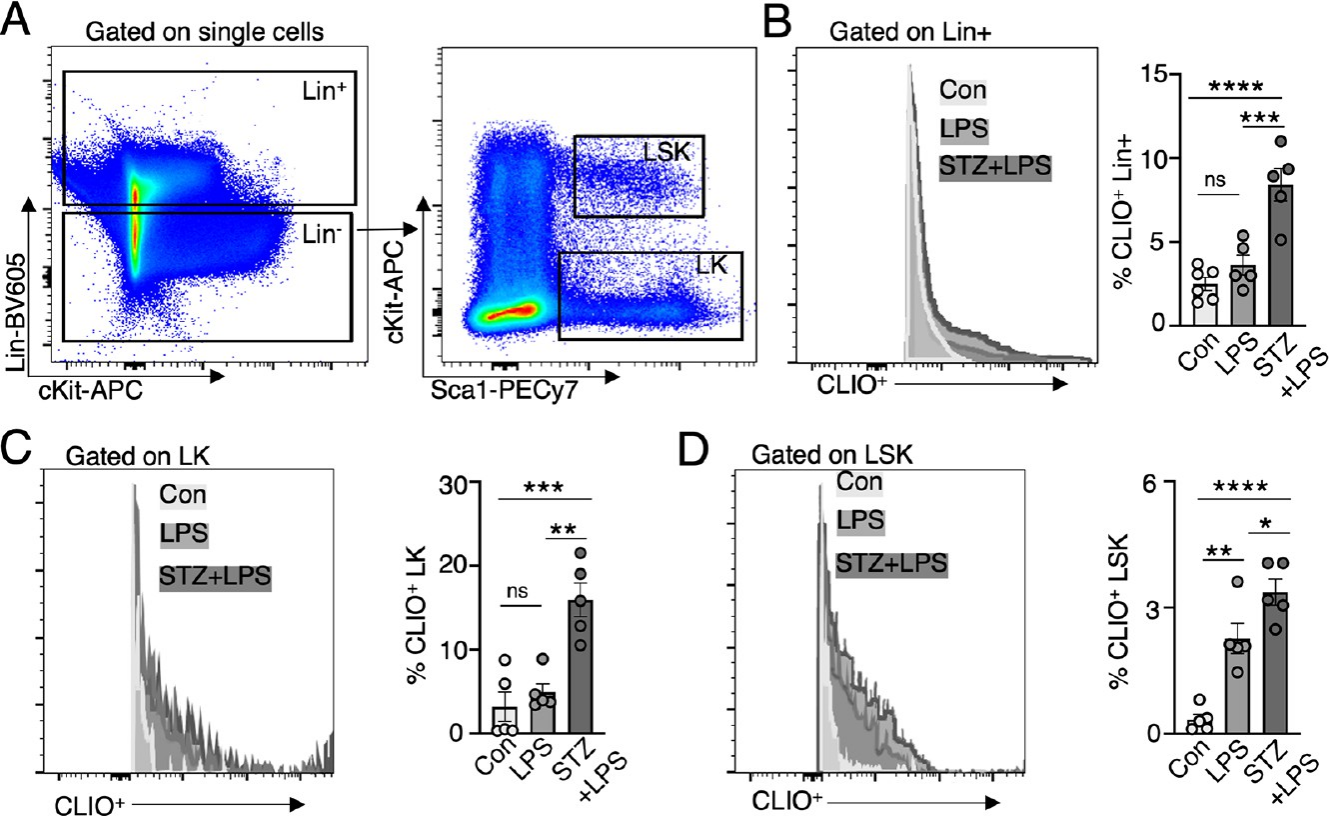
Elevated CLIO-AF647 uptake in hematopoietic stem and progenitor cells at inflamed bone marrow. (A) Gating strategy for quantification CLIO-AF647 particle uptake by Lineage^+^ (Lin^+^). From Lineage^–^ cells (Lin^–^), LSKs were identified as Lin^–^Sca1^+^cKit^+^ and LK cells were identified as Lin^–^cKit^+^. (B–D) Flow cytometry and flow cytometric quantification of CLIO^+^ uptake in bone marrow cells: (B) nonhematopoietic lineage^+^ (Lin^+^) cells, (C) Lin^–^cKit^+^ (LK), which are erythro-myeloid progenitor cells, and (D) Lin^–^Sca1^+^cKit^+^ (LSK) hematopoietic stem cells (each dot represents one mouse; *P < 0.05, **P < 0.01, ***P < 0.001, ****P < 0.0001).

In response to LPS injection, a well-characterized (Toll-like receptor) TLR ligand binds to TLR4, and elevates cell proliferation of hematopoietic stem and progenitor cells at the hematopoietic bone marrow, a process referred to as emergency hematopoiesis.^9,28^ In order to investigate which bone marrow cells take up CLIO-AF647 nanoparticles during inflammation, we conducted flow cytometry analysis on subsets of bone marrow cells in the inflamed hematopoietic bone marrow. Specifically, we examined lineage^+^ (Lin^+^) nonhematopoietic bone marrow cells, Lin^–^cKit^+^ (LK) erythro-myeloid progenitor cells, and Lin^–^Sca1^+^cKit^+^ (LSK) immature hematopoietic stem cells (Figure 7A). For Lin^+^ mature bone marrow cells and erythro-myeloid progenitor LK cells, the percentage of CLIO-AF647 uptake was notably higher in the STZ+LPS marrow, compared to the Lin^+^ and LK cells in LPS and control marrow (Figure 7B–D). Percentagewise the erythro-myeloid progenitor LK cells exhibited the highest CLIO-AF647 uptake of all bone marrow cells (Figure 7D). For more immature hematopoietic stem cells (LSKs), the percentage of CLIO uptake in LPS and STZ+LPS was significantly increased compared to that in control LSK cells (Figure 7D). These findings demonstrate that during inflammation, hematopoietic stem and progenitor cells partly take up CLIO nanoparticles in the hematopoietic marrow, labeling in particular the less mature LK hematopoietic progenitor cells, that give rise, among others, to innate immune cells.

### CLIO Nanoparticles Gradually Slow down and Become Engulfed by Innate Immune Cells in the Bone Marrow As Inflammation Levels Increase

**Figure 8:**
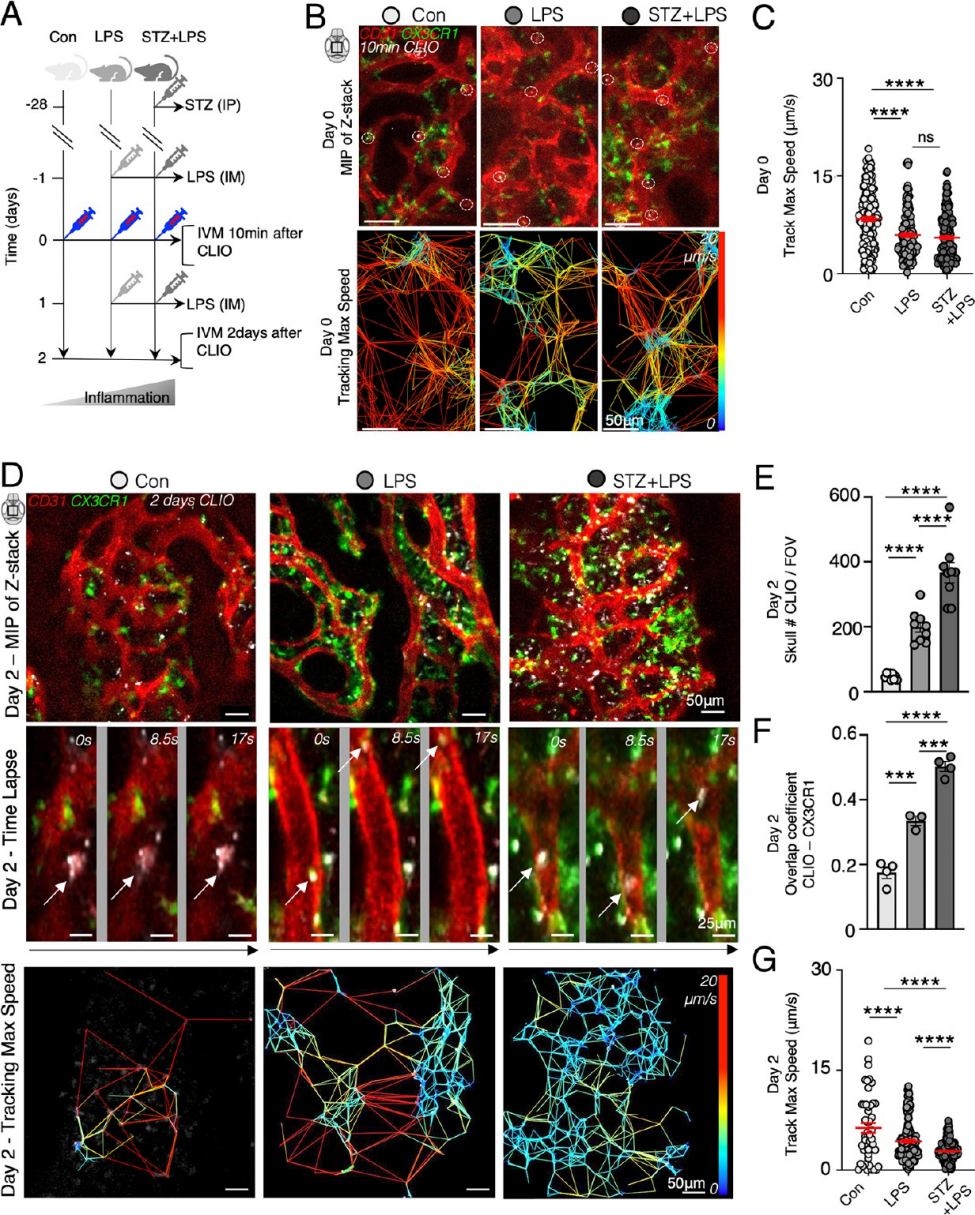
Enhanced trafficking of CLIO-AF647 nanoparticles at the bone marrow under cumulative inflammatory conditions. (A) Experimental setup illustrating that intravital microscopy (IVM) was conducted either 10 min (day 0) or on day 2 following the intravenous (i.v.) injection of CLIO-AF647 in control mice (con), mice after intramuscular (i.m.) injections of lipopolysaccharide (LPS), or in diabetic mice that also received i.m. injections of lipopolysaccharide (streptozotocin; STZ+LPS). (B) Intravital images displaying a maximum intensity projection (MIP) of a Z-stack from the calvaria 10 min post CLIO-AF647 injection i.v. Cx3cr1^GFP/+^ monocytes (green), CD31^+^ vessels (red), and CLIO-AF647 particles (white; dotted rings) are visualized. Time-lapse image processing reveals color-coded maps indicating the maximum speed of each CLIO-AF647 particle. (C) Quantification of the maximum speed of CLIO-AF647 particles 10 min postinjection. (D) Intravital images from the calvaria on day 2 post i.v. injection of CLIO-AF647 showing Cx3cr1^GFP/+^ monocytes (green), CD31^+^ vessels (red), and CLIO-AF647 particles (white), with time-lapse series of CLIO-AF647 particles and color-coded maps indicating each particle’s maximum speed. (E) Quantification of CLIO-AF647 particles in calvaria (skull marrow) per field of view (FOV). **(F**) Quantification of the overlap coefficient between CLIO and Cx3cr1^GFP/+^ monocytes in the calvaria. (G) Quantification of the maximum speed while tracking each CLIO-AF647 particle 2 days after injection.

We utilized intravital confocal microscopy to observe the calvaria at two time points: 10 min and 2 days after the intravenous injection of CLIO-AF647 in Cx3cr1^GFP/+^ control, LPS, and STZ+LPS mice. This approach allowed us to monitor the location of CLIO-AF647 relative to Cx3cr1^GFP/+^ monocytes and CD31^+^ blood vessels at the bone marrow in vivo (Figure 8A). Tracking CLIO-AF647 nanoparticles in real-time on day 0, just 10 min after intravenous injection, revealed their presence within the vessels of the skull’s marrow (Figure S4A). After 10 min, we observed a significant deceleration and clustering of particles on the vessels within the hematopoietic niche in the calvaria of LPS and STZ+LPS-treated mice. This suggests that the inflamed bone marrow acts as a storage site for nanoparticles immediately after they are injected. However, at day 0, the particles had not yet colocalized with Cx3cr1^GFP/+^ monocytes (Figure 8B, C; Figure S4A; Movies S1–S3).

On day 2 following CLIO-AF647 administration, real-time imaging of CLIO-AF647 nanoparticles, Cx3cr1^GFP/+^ monocytes, and CD31^+^ vessels showed an increased accumulation of CLIO in the skull’s marrow (Figure 8D, E; Figure S4B; Movies S4–S6). Furthermore, the highest colocalization of CLIO-AF647 nanoparticles with Cx3cr1^GFP/+^ monocytes was observed in STZ+LPS skull’s marrow, as compared to control and LPS skull’s marrow (Figure 8F). Similar to the day 0 results, the clustered CLIO-AF647 nanoparticles in the skull’s marrow of LPS and STZ+LPS mice exhibited slower trafficking than CLIO-AF647 nanoparticles in the marrow of control mice. By this time point, the majority of CLIO-AF647 nanoparticles within the blood vessels had been taken up by Cx3cr1^GFP/+^ monocytes (Figure 8F, G; Figure S4B; Movies S4–S6). These findings demonstrate a more substantial uptake of the deposited nanoparticle pool by Cx3cr1^GFP/+^ monocytes in the skull’s bone marrow as the inflammation levels increase.

## Discussion

This research delineates the enhanced retention of CLIO-AF647 nanoparticles within the bone marrow, correlating with increased levels of inflammation. Optimizing a dual imaging technique that combines R2* MRI and fluorescence imaging, we observed that macrophages effectively engulfed CLIO nanoparticles. Flow cytometry confirmed in vivo uptake of CLIO-AF647 nanoparticles by blood myeloid cell subsets, such as monocytes and neutrophils. As the degree of inflammation heightened, there was a corresponding increase in the uptake of nanoparticles in the inflamed bone marrow, which aligned with the severity of inflammation. We further present initial evidence of an elevated level of nanoparticle accumulation in the inflamed bone marrow, an effect largely attributed to the EPR effect. Additionally, our findings indicate a greater affinity of nanoparticles for hematopoietic stem and progenitor cells under inflammatory conditions. Intravital microscopy performed shortly after injection disclosed a significant slowdown of CLIO-AF647 nanoparticles in the vascular niches of the inflamed bone marrow. This effect became more pronounced at 2 days after CLIO-AF647 administration, as we observed an increased colocalization of nanoparticles with slowly patrolling Cx3cr1^GFP/+^ monocytes within the inflamed bone marrow niches. In conclusion, our results provide robust evidence for the bone marrow’s role as a distribution center for nanoparticle transport, particularly emphasizing its interaction with hematopoietic progenitor cells and their myeloid offspring during inflammation.

Our study expands on previous investigations that focus on employing iron oxide nanoparticles to monitor the migration of innate immune cells to inflamed areas across various disease settings.^1−5,16,29^ Previous investigations have predominantly linked the biodistribution of these nanoparticles and their uptake by innate immune cells to tissues such as the blood, spleen, lymph nodes, liver and bone marrow.^16−18^ Although it is known that innate immune cells efficiently ingest nanoparticles to demarcate inflammation areas, the specifics of how iron oxide nanoparticles are internalized by different cell types and trafficked during inflammatory states have not been fully delineated. Our research began with validating and measuring the effective labeling of CLIO-AF647 nanoparticles by macrophages in vitro. In accordance with previous studies,^30^ we found that CLIO-AF647 nanoparticles fuse with lysosomes when internalized by macrophages. We further optimized the detection capabilities of the MRI and fluorescence imaging modalities. R_2_* mapping surfaced as the most sensitive approach, aligning with findings from other studies.^17,33^ Two days post intravenous administration, in vivo CLIO-AF647 nanoparticles were ingested by circulating myeloid cells, including monocytes and neutrophils.^1^ Although macrophage polarization upon iron oxide nanoparticle ingestion has been previously reported,^31,32^ our observations did not reveal a change in immune profile in healthy mice.

Two days after intravenous injection, we observed minimal presence of CLIO-AF647 nanoparticles in the muscle of healthy control mice, as quantified by ΔR_2_*. In contrast, mice that received two intramuscular injections of lipopolysaccharide exhibited a pronounced uptake of these nanoparticles in the inflamed muscle. An even greater uptake of CLIO-AF647 nanoparticles in the muscle was noted when mice had streptozotocin-induced diabetes in addition to lipopolysaccharide injections. Hence, our MRI data reliably demonstrated incremental variations in R_2_* that correlated with rising inflammation levels, thereby acting as a marker for local inflammation in the muscle. These findings were confirmed by both in vivo and ex vivo fluorescent imaging and align with studies that employed iron oxide nanoparticles to trace innate immune cells at sites of inflammation.^1−5^

Our study indicates that an increase in inflammation within muscle tissue correlates with a higher uptake of nanoparticles by bone marrow. This finding was documented through both in vivo R2* mapping and fluorescent IVIS imaging techniques. While at first this may appear contradictory—considering that inflammation typically prompts a release of innate immune cells from the bone marrow^9,34^—this phenomenon is in fact consistent with the established dynamics of iron recirculation to the erythroid marrow. The uptake of nanoparticles at the bone marrow under normal circumstances has been described in other studies employing iron oxide nanoparticle-enhanced imaging.^17,35,36^ This enhanced bone marrow uptake during inflammation is actually in alignment with the recognized patterns of iron distribution, which include the recycling of iron back to the erythroid marrow where red blood cells are formed—a process that is part of the body’s normal response to inflammation.^37^ Additionally, the reticuloendothelial system, a network of cells and tissues present in the spleen, liver, and bone marrow that is responsible for the sequestration of iron, plays a significant role in this context.^38^ It captures and stores iron as part of the body’s defense mechanism, holding it back from pathogens during an inflammatory response and influencing the distribution and retention of nanoparticles.^37^

Our data suggest that the enhanced uptake of nanoparticles in the inflamed bone marrow resulted from increased permeability. This assertion is supported by our pre-CLIO contrast-enhanced MRI scans of the bone marrow. Prior studies have shown how particle extravasation occurs through diaphragmatic fenestrations in the endothelial lining of bone marrow sinusoids.^39^ Our previous research also highlighted a significant increase in permeability and retention in bone marrow during episodes of acute systemic inflammation.^9^ These EPR effects are known to correlate strongly with nanoparticle size.^29^ The EPR effect has specifically been characterized in tumor tissue vasculature, which, unlike many other vascular beds, is characterized by an extensive network of newly formed blood vessels, and an imperfect vascular architecture.^40^ The bone marrow endothelium, akin to that of tumor vessels, displays enlarged intercellular spaces, which facilitates the trapping of nanoparticles, such as the 76.54 ± 4.00 nm CLIO particles utilized in our experiments, through EPR effects.^9,40^ Furthermore, the removal of circulating nanoparticles by the reticuloendothelial system—which includes the liver, spleen, and bone marrow with their fenestrated endothelial cells—has been documented.^5^

In the context of inflammation, there is a complex interplay at the bone marrow between the release of immune cells into circulation and the activation of the hematopoietic system to replenish these cells, ensuring equilibrium during inflammatory events.^41^ Our research reveals that mature lineage-positive (Lin^+^) bone marrow cells, which have differentiated into specific cell types including various innate immune cells,^42^ showed increased per-cell uptake of CLIO nanoparticles, as reflected by median fluorescence intensity in flow cytometric analysis.

Additionally, our findings indicated a marked rise in nanoparticle absorption by hematopoietic stem and progenitor cells—specifically LSK and LK cells—in proportion to inflammation severity. LSK cells, being undifferentiated and closer to the true hematopoietic stem cell population,^42^ along with LK cells, which are precursors to myeloid lineages, showed internalization of nanoparticles during inflammatory responses. This uptake by hematopoietic progenitors, which is essential for generating innate immune cells, underscores the pivotal role of these cells in the nanoparticle internalization process during inflammation. Significant nanoparticle uptake was also noted in the less mature LK cells, which are instrumental in generating myeloid progenitors and thereby bolstering the innate immune response during inflammation.^42,43^ These results underscore the potential of iron nanoparticles as precision drug delivery systems, particularly aimed at modulating myeloid progenitors and their descendants within the immune system. Leveraging this uptake mechanism could allow for the direct delivery of therapeutic agents to these critical cells, potentially increasing the effectiveness and specificity of treatments for diseases where myeloid progenitor cells are key players. The findings indicate that the phenotypic alterations induced by iron nanoparticles on macrophages might extend to hematopoietic stem and progenitor cells within the bone marrow. Specifically, ferumoxytol, an iron nanoparticle formulation approved for clinical use, could influence the hematopoietic process, potentially altering the development of myeloid cells right from their genesis in the bone marrow, which could result in the immunomodulatory effects that have been described.^32,44^

Intravital microscopy demonstrated the real-time deceleration and accumulation of CLIO nanoparticles in the inflamed bone marrow, occurring as quickly as 10 min post intravenous injection in Cx3cr1^GFP/+^ mice. Prior research has documented the gathering of iron-containing granules in proximity to sinusoidal endothelial cells.^45^ The increased deceleration rate of particles during inflammation could be due to the bone marrow vascular niche’s role in processing cellular waste,^37^ such as aged red blood cells, atypical erythroblasts, and expelled nuclei.^45^ This process plays a crucial role in recycling and delivering iron, ultimately ensuring the production of erythrocytes.^46,47^

Two days after CLIO injection, Cx3cr1^GFP/+^ monocytes were observed engulfing nanoparticles and demonstrating diminished mobility within the inflamed bone marrow. This reduction in monocyte movement has been previously observed in activated endothelium in other vascular beds.^48^ A limitation of this study is the incomplete understanding of the mechanisms behind the reduced velocity of the Cx3cr1^GFP/+^ monocytes. A hypothesis is that these monocytes may adhere to specific cell surface receptors, such as ICAM-1 or VCAM-1, initiating signaling cascades that result in alterations to the cellular machinery and cytoskeleton. These changes can temporarily slow down or halt cell movement, resulting in a temporary alteration of cell dynamics and motility.^48^ Overall, our findings uncover the bone marrow’s enhanced ability to sequester and redistribute nanoparticles in the presence of increased inflammation.

## Conclusions

Here we implemented iron oxide nanoparticle imaging of the bone marrow, providing a valuable tool for tracking different levels of inflammation. Consequently, iron oxide nanoparticles can serve as an imaging biomarker to gauge the severity of inflammatory diseases and visualize inflammation and immune cell responses in various conditions, including cardiovascular disease, cancer, to neurodegenerative diseases. This research underscores the potential of targeted delivery of iron oxide nanoparticles directly to immune cells by the hematopoietic bone marrow, highlighting its therapeutic implications. Our findings address the critical need for clinical indicators of inflammatory activity in multiple diseases, acting as prognostic markers for disease outcomes and benchmarks for the assessment of future immunomodulatory treatments.

## Methods

### CLIO-AF647 Nanoparticles

Dextran cross-linked iron oxide (CLIO) magnetic nanoparticles fluorescently labeled with AF647 (CLIO-AF647; 40 kDa; Luna NanoTech, Markham, ON, Canada; 5 mg/mL) were characterized by dynamic light scattering (DLS), assessing their size distribution and zeta potential. Measurements were carried out with a Zetasizer Nano ZS from Malvern Panalytical Ltd., Malvern, United Kingdom. The data from the autocorrelation analysis were refined by using the Contin algorithm in the Zetasizer software (version 7.13). Measurements for particle size and zeta potential were conducted using dilutions of 1:100 in double distilled water and 1:20 in saline, respectively, with each set performed in triplicate.

### Animals

C57BL/6J (from Envigo, Israel) and B6.129P-Cx3cr1tm1Litt/J (Cx3cr1^GFP/+^; a kind gift from Prof. Steffen Jung, Weizmann Institute, Israel) female mice 8–12 weeks old were randomly allocated to the experimental groups. The Cx3cr1^GFP/+^ mouse is widely used for studies of the mononuclear phagocyte system.^26^ The CX3CR1 locus had been replaced with an eGFP construct (knock-in), resulting in cytosolic GFP fluorescence.^26^ In Cx3cr1^GFP/+^ heterozygotes, several myeloid lineages are GFP+ including monocytes, dendritic cells, tissue-resident macrophages, brain microglia, and subsets of NK and T cells.^26^ All applicable protocols, animal handling, surveillance, and experimentation were performed in accordance with the Technion Institutional Animal Care and Use Committee.

### Cells

RAW264.7 macrophages were purchased from ATCC and cultured in medium consisting of DMEM, fetal bovine serum (FBS) 10%, l-Glutamine 1%, and penicillin/streptomycin 1% (all from Sigma). To obtain primary bone marrow derived macrophages (BMDM), healthy C57BL/6J mice (n = 6) were sacrificed, and bone marrow cells were flushed with DMEM from the femur and tibia and isolated through a 70-μm mesh. After osmotic erythrocyte lysis, cells were pelleted, counted, and resuspended in BMDM differentiation medium, which included the same medium with the addition of 5 ng/mL M-CSF (Peprotech) for 7 days.

### In Vitro Phagocytosis

RAW264.7 macrophages or BMDMs were detached from culture plates by incubation with 0.05% Trypsin-EDTA for 5 min. N = 1 × 10^6^ cells were incubated with 200 μL solution in Eppendorf tubes for 2 h at 37 °C with 10 mg/mL contrast agent. The incubation solution included 30 μL FBS, 70 μL medium with cells, 40 μL CLIO-AF647 (5 mg/mL) and 60 μL PBS. After incubation, the cells were washed five times. To estimate cell viability, a Trypan Blue viability assay was done counting the number of stained (dead) and unstained (viable) cells using a hemocytometer after incubation with Trypan Blue. To evaluate the phagocytic ability, we followed the uptake of CLIO-AF647. For target-to-background analysis (TBR) of fluorescent microscopy, RAW264.7 macrophages were imaged in a 96-cell plate by a LionHeart microscope (BioTek) with a 10 ms exposure, 10% digital gain, 100% LED intensity and image-based autofocus. For immunocytochemistry of BMDM, coverslips with adherent CLIO-incubated macrophages were fixed and with 4% PFA for 20 min. To block unspecific antibody binding, for 30–60 min in BSA (1–3%) solution in PBS. Next, F4/80 antibody (1:400; rat antimouse; catalog no.14–4801–81; eBioscience) was used overnight at 4 °C to verify macrophage differentiation. To detect the primary antibody, an antirat secondary antibody labeled with Alexa Fluor 546 (catalog no. A11081; Thermofisher) was used. After staining, cells were washed and mounted with DAPI mounting medium. To probe lysosomal uptake, RAW264.7 macrophages were first incubated for 1h with Lysotracker (Lysotracker Red DND-99; catalog no. L7528; Thermofisher) and after incubated with CLIO nanoparticles as described above. Confocal microscopy was done on a confocal inverted confocal microscope (Zeiss LSM700). ImageJ software was used to calculate TBR.

### Flow Cytometry

For flow cytometric analysis of blood myeloid cells, blood was isolated from the heart of healthy mice with or without CLIO administration 48h prior. Consequently, red blood cells were lysed with a red blood cell lysis buffer (420301, Biolegend). The remaining cells were stained with CD45-BV711 (clone 104), CD11b-BV421 (clone M1/70), Ly-6C–BV605 (clone HK1.4), and Ly-6G-PECy7 (clone 1A8) (all 1:1500 dilution, BioLegend). After staining, samples were fixed with paraformaldehyde and acquired on an LSR Fortessa flow cytometer (BD Biosciences). To ensure data integrity, cell doublets were excluded via forward scatter width (FSC-W) versus forward scatter area (FSC-A) analysis, and dead cells were identified and removed based on their diminished size and low granularity in the side scatter area (SSC-A) versus FSC-A plot. Myeloid cells were identified based on their CD45^+^CD11b^+^ expression, Ly6C^high/intermediate^ (Ly6C^high/int^) monocytes, Ly6C^low^ monocytes, and Ly6G^+^ neutrophils were differentiated according to the Ly6C and Ly6G expression.

For flow cytometric analysis of BMDM, CLIO-incubated BMDM were stained to detect macrophage differentiation with the following antibodies: APC.eFluor780-labeled F4/80 AB (catalog no. 47–4801–50; eBioscience). To detect and quantify labeling of CLIO-incubated BMDM, cells were visualized by an ImageStream^X^ MkII imaging cytometer, 60× magnification; with low flow rate/high sensitivity using INSPIRE software. The percentage of CLIO^+^ BMDM was measured for labeled (2h CLIO incubation) and unlabeled cells (incubation without CLIO). For flow cytometric analysis to differentiate the cell-types of CLIO-AF647 nanoparticle uptake in the bone marrow, mice 2 days after CLIO injection were sacrificed, and femurs were dissected. All single-cell suspensions were treated with red blood cell lysis and stained at 4 °C in 300 μL of FACS buffer (PBS supplemented with 0.5% BSA). For HSPC staining, isolated bone marrow cells were first stained with an antibody cocktail containing CD5, CD45R(B220), CD11b, Anti–Gr-1, (Ly-6G/C),7–4 and – Ter-119 (1:300 dilution, Miltenyi Biotec). This was followed by a second staining with antibodies for c-kit-PE/Cy7 (105814, clone 2B8), Sca-1-BV605 (108133, clone D7) and streptavidin-APC/Cy7 (405208; all 1:300 dilution, BioLegend). Cytek Aurora is flow cytometer equipped with four lasers (405, 488, 561, 635 nm) was used to detect and quantify labeling of CLIO. Lineage^+^ were identified as Lin^+^. From Lineage^–^ cells (Lin^–^), LSKs were identified as Lin^–^Sca1^+^c-Kit^+^ and LK cells were identified as Lin^–^cKit^+^. Flow cytometry data analysis was performed in Flowjo (v10.7.1, BD Biosciences).

### Phantom Preparation

Two phantoms were prepared. The first phantom consisted of various concentrations of CLIO-AF647 with agarose in Eppendorf tubes of 200 μL, filled with 2% agarose gel in PBS using different concentrations (0.001–0.003 mM) and left at room temperature to solidify. The second phantom contained various numbers of CLIO-AF647-incubated RAW264.7 macrophages (between 2500 and 100 000 cells) were prepared in a total volume of 100 μL of 2% agarose in PBS in 200 μL Eppendorf tubes. Pure agarose gel (100 μL) and unstained cells in agarose were used as the baseline in all the measurements. The two phantoms were subjected to MRI and IVIS imaging to validate the uptake of CLIO-AF647 by RAW264.7 macrophages.

### Mouse Inflammation Model

To image increasing levels of inflammation, mice were randomly divided into 3 groups: 1) Control (Con) healthy mice injected with CLIO-AF647 at day 0 at 12-week-old; 2) Mice were injected with lipopolysaccharide (LPS; 100 ng from *Escherichia coli* O55:B5, Sigma-Aldrich)^49^ in the left vastus lateralis muscle (i.m.) twice (1 day before and 1 day after i.v. CLIO injection); 3) Mice with induced type 1 diabetes at 8 weeks of age by IP injections of streptozotocin (STZ+LPS; 50 mg/kg in NaCitrate solution, Sigma-Aldrich) on 5 consecutive days (±28 days before CLIO). In addition, diabetic mice were injected twice with lipopolysaccharide i.m. (in the vastus lateralis muscle) 1 day before and 1 day after CLIO). The body weight was 20 ± 2 g in each mice group at 8 weeks of age. Glucose levels of diabetic mice were closely monitored. CLIO-AF647 (15 mg/kg) was iv injected at Day 0, immediately after the pre-CLIO MRI.

### MRI Imaging

For phantom imaging, MRI was used to calculate the relaxivity (*r*_1_, *r*_2_, and *r*_2_*), which is the degree to which the CLIO nanoparticles can enhance the longitudinal or transverse water relaxation rate constant (*R*_1_ = 1/ *T*_1_, *R*_2_ = 1/*T*_2_, and *R*_2_* = 1/*T*_2_*) normalized to concentration of CLIO nanoparticles. MR imaging was performed on a horizontal bore 9.4T MRI system (Bruker Biospec; Ettlingen, Germany) with a cylindrical volume coil (86 mm inner diameter) for RF transmission and detection. Four different protocols for *T*_2_-weighted, *T*_1_, *T*_2_, and *T*_2_* mappings were used. All images were acquired with the same geometry and resolution: slice thickness 0.7 mm, field of view 8 × 8 cm, matrix size 256 × 256, total of 2 slices. *T*_2_-weighted images were acquired with a Rapid Acquisition with Relaxation Enhancement (RARE) sequence with the following parameters: repetition time (TR): 2.5 s, echo time (TE): 30 ms, RARE factor: 4, averages: 10 4. For *T*_1_ mapping, a RARE sequence was performed with variable TRs: 250, 400, 600, 800, 1000, 2000, 3000, 4000, 5000, 6000, 7000, 8000 ms; TE: 24.3 ms, RARE factor: 8, averages: 1. For *T*_2_ mapping, a multislice multiecho (MSME) sequence was performed with TR: 2 s and TEs: 11, 22, 33, 44, 55, 66, 77, 88, 99, 110 ms, averages: 1. For *T*_2_* mapping, a multigradient echo (MGE) sequence was performed with TR: 1.5 s and TEs: 3, 6, 9, 12, 15, 18, 21, 24, 27, 30, 33, 36 ms, averages: 2. Analysis of MR imaging data was performed by using custom software in Matlab (Mathworks, Natick, Mass). Regions of interest (ROIs) were drawn on gray scale images with TE = 3 ms. Pixel-wise fitting of MR imaging data was performed using custom software in Matlab R2021b (Mathworks, Natick, MA). *R*_1_ longitudinal relaxation time was calculated by using a least-squares optimization curve-fitting algorithm: SI(TR) = SI_0_[1 – *Ce*^–TR*R*_1_^)]. *R*_*n*_ = *R*_*2*_ and *R*_2_* transversal relaxation times were quantified by fitting: SI(TE) = SI_0_*e*^–*R_n_*TE^ + *C*, where SI_0_ is signal intensity with an echo time of 0 ms; *R*_*n*_ is the transverse relaxation rate, and *C* is the noise at the last echo time. The relaxivity (*r*_*n*,_ with *n* = 1, 2 ,or 2*) of the CLIO nanoparticles was defined as the change in relaxation times (*R*_*n*_, with *n* = 1, 2, or 2*), per concentration of the particles (*C*_CLIO_): *r*_*n*_ = (*R*_*n*_ – *R*_*n*_^0^)/*C*_CLIO_. Thus, relaxivity of the CLIO particles is the degree to which they can enhance the longitudinal or transverse water relaxation rate constant (*R*_1_ = 1/*T*_1_ or *R*_2_ = 1/*T*_2_, respectively) normalized to the concentration of the CLIO nanoparticles. All phantom experiments were conducted in triplicate.

In vivo MRI was performed on a horizontal bore 9.4T MRI system (Bruker Biospec; Ettlingen, Germany), using a cylindrical volume coil (86 mm inner diameter) for RF transmission and a surface coil (20 mm diameter) for detection. Animals were anesthetized (induction 3%; maintenance 1.25% in oxygen (0.7 L/min)) and positioned on the right side while warmed by using circulating hot water, and their respiration rate was monitored during imaging (Small Animal Instruments, Stony Brook, NY). To define bone marrow boundaries, mice were injected with IP with 0.2 mmol/kg gadoterate meglumine (Dotarem; Gd-DOTA), to assess the perfused area of the bone marrow. A MGE pulse sequence was used to acquire a series of *T*_2_* images with TR: 1.5s /, first TE: 3 ms, echo spacing: 3 ms 3 and total of 12 echoes 12. Three slices with slice thickness 0.7 mm, field of view 4 × 4 cm, matrix size 256 × 256, 156 μm in plane resolution, averages: 2. For *T*_1_ mapping, a RARE VTR sequence was performed with the same geometry and resolution as the MGE, and with variable TRs: 250, 400, 600, 800, 1000, 2000, 3000, 4000, 5000, 6000, 7000, 8000 ms, TE: 24.3 ms, RARE factor: 8, averages: 1. Analysis of MR imaging data was performed as described above.

### Fluorescence Imaging

For phantom fluorescence imaging, phantoms were placed inside a preclinical IVIS (IVIS SpectrumCT, PerkinElmer) and fluorescence intensity was recorded using the bandpass emission wavelength of 680 nm and excitation wavelength of at 640 nm. The fluorescence intensity, scale bars, and color maps were adjusted to the same levels.

For whole body fluorescence imaging, mice were imaged using the same IVIS instrument (PerkinElmer IVIS Spectrum CT) at day 2 post-CLIO. Prior to imaging, mice were shaved and depilated to remove all hair that otherwise absorbs light and interferes with optical imaging. Mice were anesthetized (Isoflurane 1.7%, O_2_ 2 L/min). Mice were imaged to detect the specific fluorescence of CLIO-AF647. The fluorescence intensity, scale bars and color maps were adjusted to the same levels. For ex vivo quantification of fluorescence intensity, the femur, muscle, liver, spleen, and kidney were dissected and imaged to detect specific fluorescence of CLIO-AF647. Fluorescence IVIS imaging data were analyzed with Live Image 4.5.4 software.

### In Vivo Intravital Microscopy

Intravital microscopy (IVM) was performed in vivo on calvaria (skull marrow) of *n* = 2 mice per group using a confocal Zeiss 880 microscope with an upright Axio Examiner Z1 stand. Per mouse n = 3–5 different regions of interest were imaged at the calvaria. First, 10 min or 2 days after iv CLIO-AF647 injection, Cx3cr1^GFP/+^ mice were shaved at the skull and held in a stereotaxic skull holder. Next, a skin incision revealed the calvaria, and PBS (Sigma-Aldrich) was applied to prevent the tissue drying prior to imaging. To outline the vasculature, we used Phycoerythrin (PE) anti- CD31 (50 μL, MEC13.3, Biolegend). The microscopy was performed in 3 channels: 633 nm laser for CLIO-AF647, 561 nm for CD31 (an endothelial cell marker), and 488 nm for Cx3cr1^GFP/+^ monocytes/macrophages with a water dipping objective (20x, NA-1.0, working distance-1.8 mm and parfocal length-75 mm) covering a field of view of 425.10 μm× 425.10 μm, at 512 × 512 pixels. Z-stack images were acquired in 2.5 μm steps. Time-lapse imaging was performed in the three channels with a time resolution of 1.89 frames per second (fps). Confocal images were analyzed using Fiji with the Trackmate plugin.

### Ex Vivo Confocal Microscopy and Histology

To analyze vascular permeability × surface area product, mice were i.v. injected albumin-RhoB (excitation/emission: 555 nm/581 nm, 82 kDa, 2.5 mg/mouse, Symo-chem, The Netherlands), which is a blood pool agent after in vivo imaging (MRI). Five minutes after i.v. injection, mice were sacrificed and processed as following: Following the imaging, muscles and femurs were transferred into 4% PFA solution for 12–18 h. For whole-organ ex vivo confocal microscopy, organs were subsequently moved into 15% sucrose solution for 6 h. Further, femurs and muscle were embedded in the OCT to preserve them for the fluorescence imaging in 4% PFA and embedded and frozen in optimal cutting temperature. Muscles were shaved and imaged near the inflamed site by spinning disk confocal microscope (CSU-W1, Nikon, 10x (Dry, NA-0.45, working distance-4 mm) and 20x (Dry, NA-0.70, working distance-2.30 mm) equipped with 4 solid lasers in Z-stacks of 10–16 μm, at 4 μm steps. Femurs were shaved 300 μm with a cryostat at −25 °C to expose the bone marrow. These long bones were then thawed and imaged by spinning disk confocal microscope (see above). For the femurs, Z-stack images were acquired at 4 μm steps, covering 40 μm depth. For histology, femurs were first decalcified using a 10% EDTA solution. Both contralateral femurs and (inflamed) muscle tissues of the vastus lateralis muscle were paraffinized. Sections of 4 μm thickness were cut and mounted on slides. Muscle sections were stained with H&E. Femur sections were stained with Perls’ Prussian Blue for iron. Positive staining for iron appeared as distinct blue deposits in the tissue sections. Maximal intensity projections of Z stacks were reconstructed and processed using Fiji. Fluorescent and histological images were processed and analyzed using Fiji.

### Statistics

Statistical analyses were performed by using GraphPad Prism software (GraphPad Software, Inc.). Results are reported as the mean ± standard error of the mean (sem). Mann–Whitney was used to compare the two groups, whereas one-way analysis of variance (ANOVA) was used to compare the three groups. One-way ANOVA test followed by a nonparametric Kruskal–Wallis test for three groups. Statistical tests and animal numbers for each graph are specified in the figure legends.

## Supporting Information Available

Additional details and data related to our research, including dynamic light scattering analysis and in vitro cytotoxicity evaluation of CLIO-AF647 nanoparticles (Figure S1); insights into blood metrics and the distribution of CLIO-AF647 nanoparticles under conditions of increasing inflammation (Figure S2); ex vivo imaging of CLIO-AF647 nanoparticles within the inflamed bone marrow of the entire femur (Figure S3), along with intravital images capturing the calvaria 10 min and 2 days after CLIO-AF647 injection (Figure S4). Lastly, (PDF)
Series of six dynamic intravital time-lapse movies illustrating the dynamic interactions between Cx_3_cr1^GFP/+^ monocytes (green), CD31^+^ vessels (red), and CLIO-AF647 particles (white): Movie S1, intravital time-lapse movie of control calvaria 10 min after CLIO-AF647 injection
Movie S2, intravital time-lapse movie of LPS calvaria 10 min after CLIO-AF647 injection
Movie S3, intravital time-lapse movie of STZ + LPS calvaria 10 min after CLIO-AF647 injection
Movie S4, intravital time-lapse movie of control calvaria 2 days after CLIO-AF647 injection
Movie S5, intravital time-lapse movie of LPS calvaria 2 days after CLIO-AF647 injection
Movie S6, intravital time-lapse movie of STZ + LPS calvaria 2 days after CLIO-AF647 injection

## Supplementary Material

nn3c11201_si_001.pdf

nn3c11201_si_002.avi

nn3c11201_si_003.avi

nn3c11201_si_004.avi

nn3c11201_si_005.avi

nn3c11201_si_006.avi

nn3c11201_si_007.avi
