# Supplementary material for: Cross-Modal Imaging Reveals Nanoparticle Uptake Dynamics in Hematopoietic Bone Marrow during Inflammation
Source: ACS Nano. 2024 Feb 12;18(9):7098–113. doi: 10.1021/acsnano.3c11201 (PMC10919094; doi:10.1021/acsnano.3c11201)
Supplement: Supplementary file 1 — nn3c11201_si_001.pdf [file nn3c11201_si_001.pdf]

Supporting Information for

Cross-Modal Imaging Reveals Nanoparticle Uptake Dynamics in the Hematopoietic Bone Marrow during Inflammation

Ashish Tiwari[1], Narmeen Haj[1], Betsalel Elgrably[1], Maria Berihu[1], Viktor Laskov[1,3], Sivan Barash[1], Shachar Zigron[1], Hagit Sason[1], Yosi Shamay[1], Shiri Karni-Ashkenazi[1], Maya Holdengreber[2], Galit Saar[2] and Katrien Vandoorne[1]

[1]Faculty of Biomedical Engineering, Technion-Israel Institute of Technology, Haifa, Israel

[2]Biomedical Core Facility, Rappaport Faculty of Medicine, Technion-Israel Institute of Technology, Haifa, Israel

[3]Third Faculty of Medicine, Charles University, Prague, Czech Republic

AUTHOR ADDRESS: Katrien Vandoorne

Faculty of Biomedical Engineering

Technion, Israel Institute of Technology

Technion City, Haifa 3200003,

Israel

Email: k.vandoorne@technion.ac.il

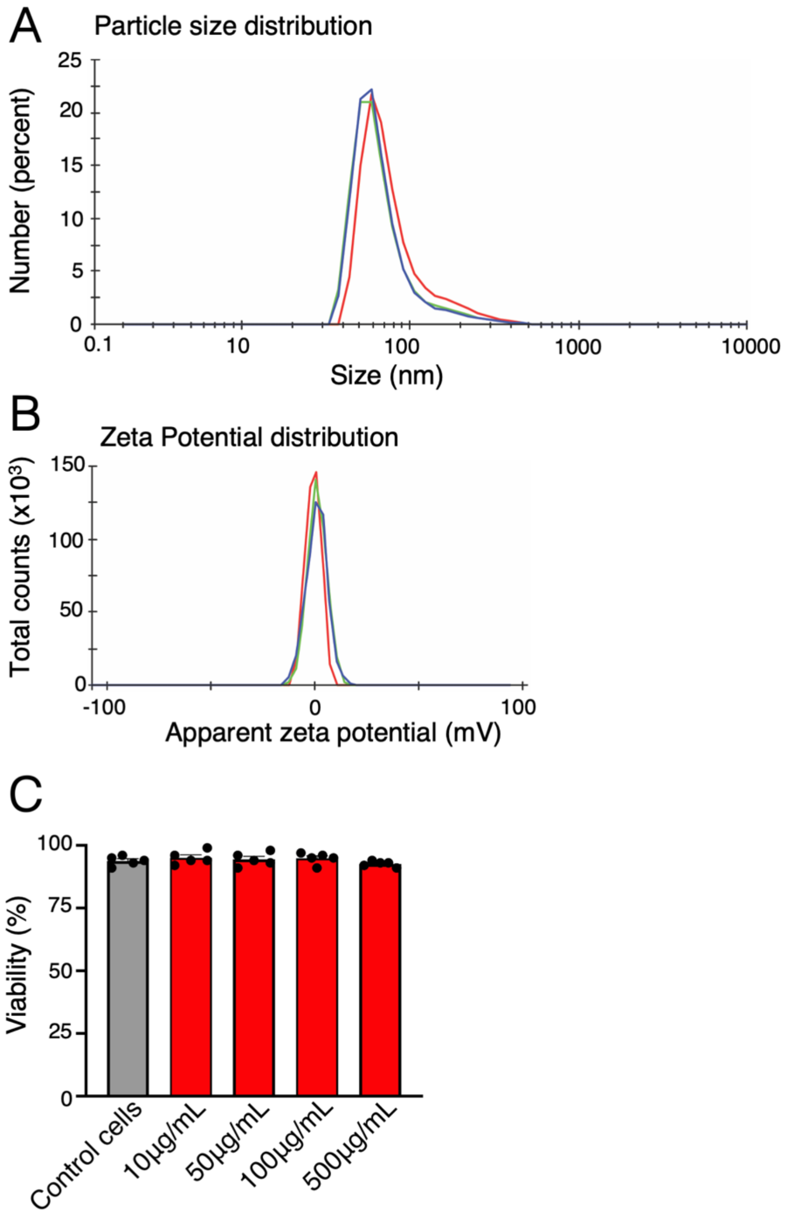

Figure S1 | Dynamic light scattering (DLS) analysis and in vitro cytotoxicity analysis of CLIO-AF647 of dextran cross-linked iron oxide (CLIO) magnetic nanoparticles fluorescently labeled with AF647 (CLIO-AF647). A) CLIO-AF647 nanoparticle size distribution and (**B**) zeta potential of CLIO-AF647 nanoparticles (CLIO-AF647; 40kDa; Luna NanoTech, Markham, ON, Canada; 5mg/mL). (**C**) Graphs depicting viability of RAW264.7 cells exposed to 2h of incremental concentrations of CLIO-AF647.

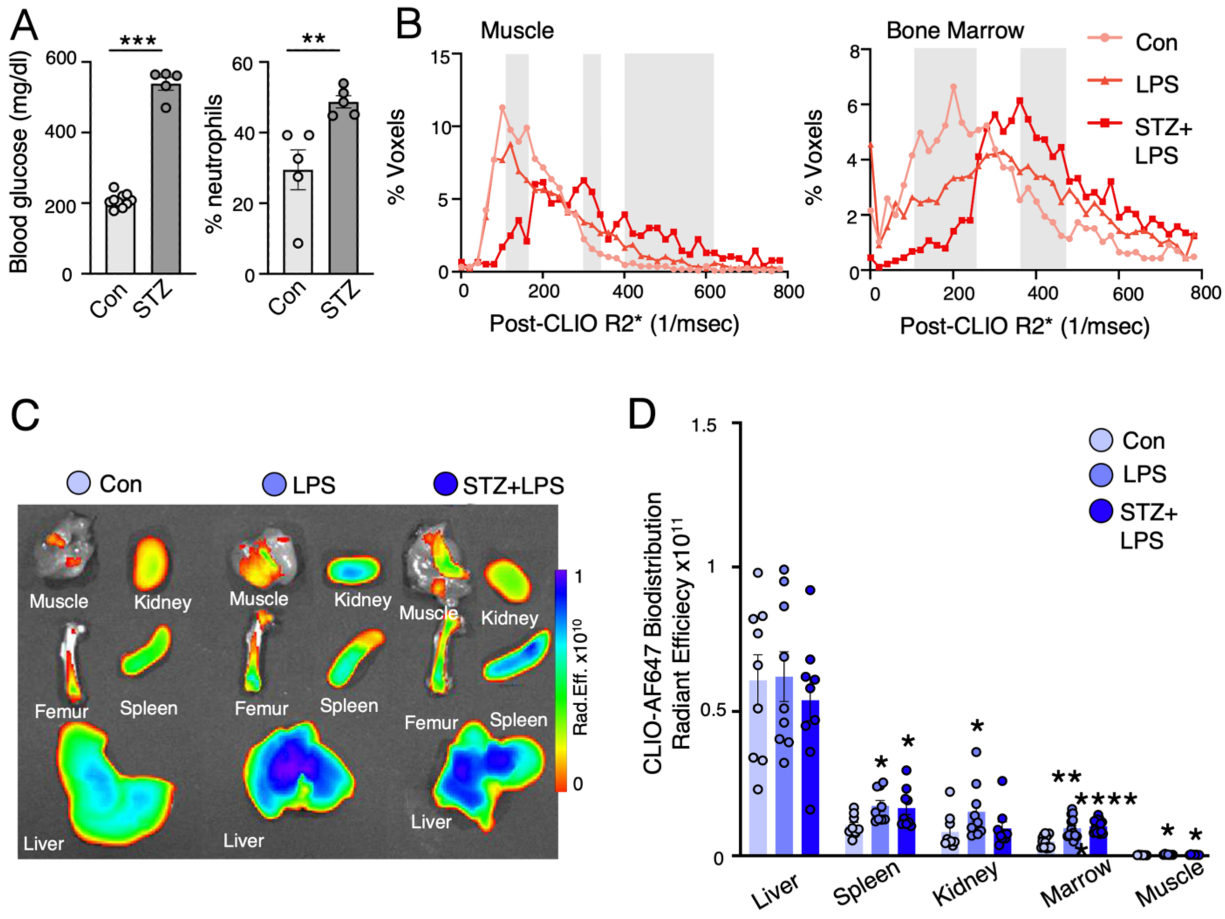

Figure S2 | Blood metrics and distribution of CLIO-AF647 nanoparticles with increasing inflammation. (**A**) Verification of glucose and neutrophil levels in diabetic mice. **(B)** Histogram analysis of the percent of voxels in each value range of R_2* in muscle and bone marrow of the femur. Post-CLIO R_2* for muscle and bone marrow averaged over the entire voxels of respectively muscle or bone marrow. Ranges with significant increase in voxels with high R_2* values in muscle and femoral marrow are indicated in grey background. (**C**) Ex vivo fluorescence imaging 2 days after i.v. injection of CLIO-AF647 nanoparticles in muscle, kidney, femur, spleen and liver. (**D**) Biodistribution of i.v. injected CLIO-AF647 nanoparticles by ex vivo fluorescence imaging.

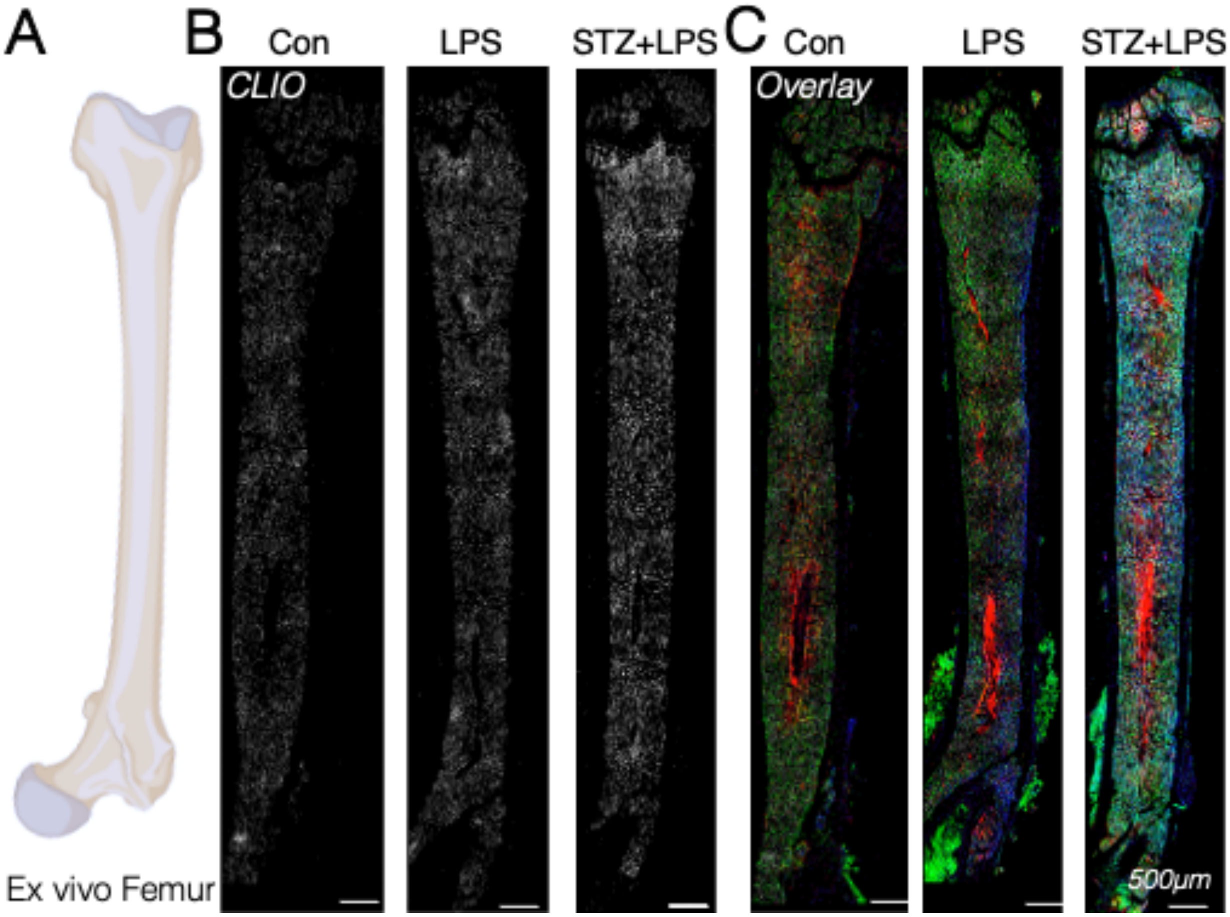

Figure S3 | Ex vivo imaging of higher levels of CLIO-AF647 nanoparticles at the inflamed bone marrow and gating strategy. (**A**) Femur outline. (**B-C**) Representative whole mount fluorescence microscopy of the femur with CLIO-AF647 particles only (**B**) and overlay (**C**) of showing $Cx_3cr1^{GFP/+}$ monocytes (green), albumin-RhoB (red), and CLIO-AF647 particles (white).

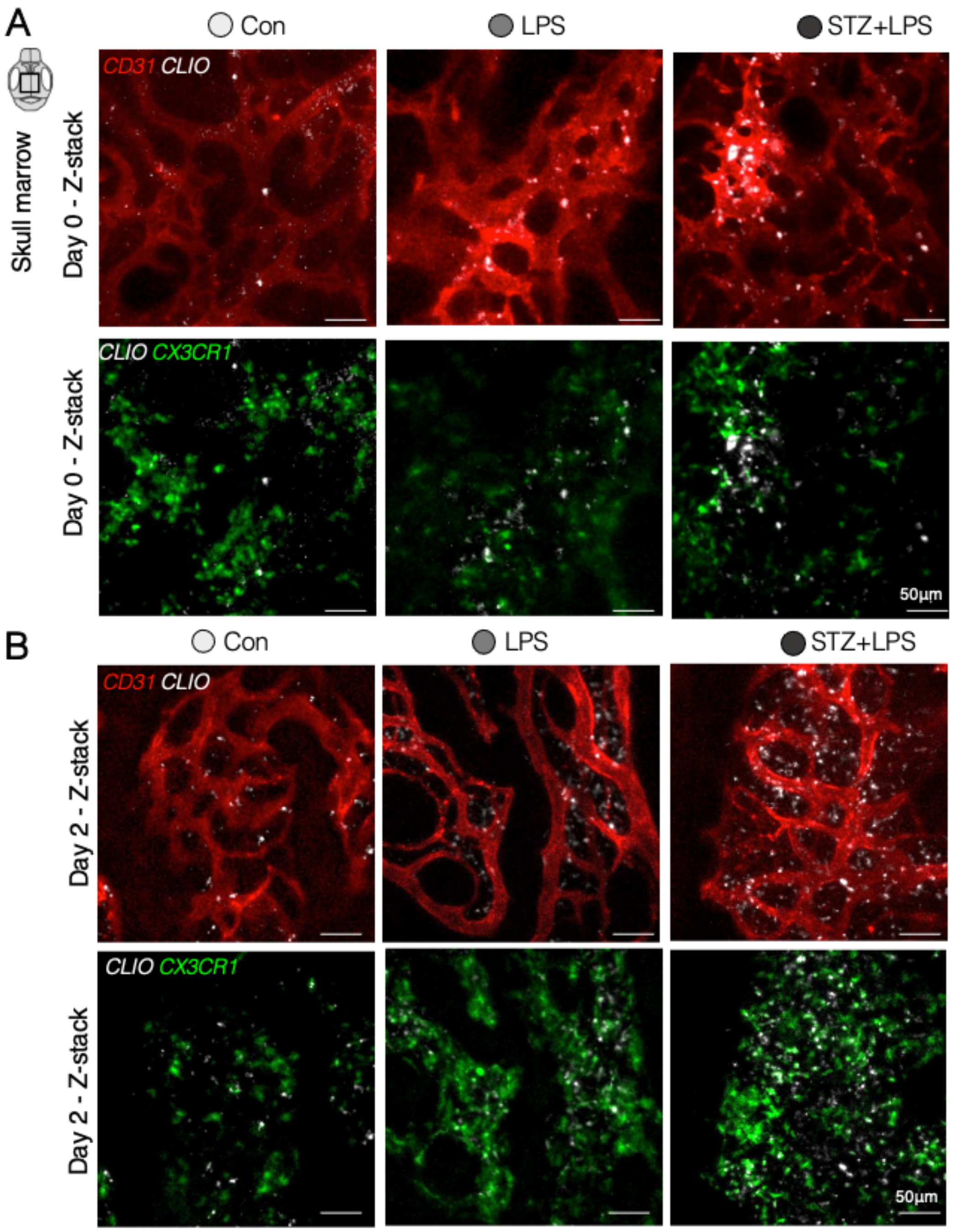

Figure S4 | Intravital images displaying a maximum intensity projection (MIP) of a Z-stack of the calvaria. (**A**) At day 0, and (**B**) at day 2 after CLIO-AF647 injection showing an overlay of $CD31^{+}$ vessels (red) and CLIO-AF647 particles (up) and an overlay of $Cx_3cr1^{GFP/+}$ monocytes (green) and CLIO-AF647 particles (down).
